# Supplementary material for: Mobile phones carry the personal microbiome of their owners
Source: PeerJ. 2014 Jun 24;2:e447. doi: 10.7717/peerj.447 (PMC4081285; doi:10.7717/peerj.447)
Supplement: Data S1 — This document details all statistical analysis and data processing reported in the manuscript. It was created using the knitr package in R. All data and associated scripts are available on GitHub: https://github.com/jfmeadow/Meadow_etal_Phones. [file peerj-02-447-s001.pdf]

# Phone Microbiome/Biosensor Project

### James Meadow (jfmeadow at gmail dot com)

---

*This analysis document accompanies a manuscript that reports analysis of microbial communities sampled from smartphone touchscreens, as well as the index fingers and thumbs of their owners. This was a project that was conducted as an educational workshop meant to explore innovative ways to monitor health. The manuscript is currently in review. This document was produced with the `knitr` package in R, and all source code can be found on GitHub: https://github.com/jfmeadow/Meadow_etal_Phones*

---

### Getting data into shape

The first step is to set a random seed (so results are locked in with those reported in the manuscript) and load some necessary R packages and functions.

```
set.seed(42)
options(scipen=7)  # curtail scientific notation
library(vegan)
```

```
Loading required package: permute
Loading required package: lattice
This is vegan 2.0-10
```

```
library(labdsv)
```

```
Loading required package: mgcv
Loading required package: nlme
This is mgcv 1.7-28. For overview type 'help("mgcv-package")'.
Loading required package: MASS

Attaching package: 'labdsv'

The following object is masked from 'package:stats':

    density
```

```
library(miscTools)
library(xtable)
library(ggplot2)

# These options just for debugging - knitr is automatically in dir.
# setwd('~/Dropbox/rwjf/Meadow_etal_Phones/')
# load('phones.RData')

source('functions.R')
```

The OTU table is brought in with a custom function `QiimeIn` that reads a classic OTU table and then splits it into a few useful pieces in a big list. The Bioconductor package `phyloseq` actually has more efficient ways to do this with the `.biom` format, but my modest function will work here - the outcome is practically the same in this case.

```
rw.list <- QiimeIn(file='phones_otu_table.txt')
# removed comment character from first line so R takes it.
rw.map <- read.delim('phones_map.txt', head=TRUE, row.names=1)
rw.big <- rw.list$Table
rw.taxo <- rw.list$Taxa
rm(rw.list)
```

Next, the OTU table needs to be put into shape. R wants to see letters, not numbers, as row names, so a big X is inserted. Remove this. We found a total of 3207836 sequences, consisting of 56 samples and 34400 OTUs, defined at 97% sequence similarity. Then line up with the order of the mapping file.

```
row.names(rw.big) <- gsub('X', '', row.names(rw.big))
rw.big <- rw.big[row.names(rw.map), ]
```

---

### Configure OTU table and check for contaminants

Before anything else, we should remove any OTUs from lab controls that showed up in experiment samples. Cell phone surfaces hold really low biomass, so amplification contamination is inevitable. Best to just remove them all. Save the list of contaminant OTUs to shore up the taxonomy table below.

To answer a question that arose during the first round of peer review, the contaminants are assessed for their influence over final results. So a table with contaminants, one minus only abundant contaminants, and one with all removed will be kept and assessed downstream.

```
cont <- grep('cont', row.names(rw.map))  # which samples are controls
cont.table <- rw.big[cont, ]  # otu table of only controls
cont.otus <- which(colSums(cont.table) >0)  # which otus are present in controls
cont.otus.names <- colnames(cont.table)[cont.otus]  # what are their names
```

```
cont3.otus <- which(colSums(cont.table)/sum(cont.table) > 0.05)  # pick out 3 big ones
rw.table.noTop3control <- rw.big[-cont, -cont3.otus]  # take out big 3
```

---

Next:

- Plant sequences are always in 16S datasets, so one way to remove them is to call them by name "*Streptophyta*." These get removed.
- Do the same for mitochondrial sequences.
- Remove lab contaminants identified above.

- Remove OTUs that are represented by only 1 or 2 sequences - these lend little to community analysis and slow down the whole works.
- The last step is to rarefy all samples to an even sampling depth, in this case 7000 sequences per sample.

```
rw.taxo <- rw.taxo[-cont3.otus, ]
streptophyta <- grep('Streptophyta', rw.taxo$taxa.names)
mitochondria <- grep('mitochondria', rw.taxo$taxa.names)
rw.table.tmp <- rw.table.noTop3control[, -c(streptophyta, mitochondria)]
rw.table.tmp <- rw.table.tmp[, -c(which(colSums(rw.table.tmp) < 3))]
sort(rowSums(rw.table.tmp))
```

```
20.phone 28.index 30.phone 35.phone 29.phone 31.phone 30.thumb 22.phone
    7357    15231    17361    18739    18996    19424    19912    21284
18.phone 26.thumb 20.thumb 30.index 34.phone 28.phone 35.index 28.thumb
   21829    22650    24056    26001    26493    27132    29177    29364
20.index 35.thumb 19.phone 18.index 25.phone 23.thumb 17.thumb 23.phone
   30595    32067    32604    39787    39989    42323    43409    44325
24.phone 18.thumb 29.index 17.phone 32.index 23.index 19.index 29.thumb
   46025    46431    47540    47730    47800    48302    49779    50515
26.phone 22.index 26.index 32.thumb 22.thumb 17.index 31.index 25.thumb
   51103    52518    57129    58278    58901    59207    60430    66851
34.thumb 32.phone 31.thumb 33.phone 25.index 33.thumb 24.thumb 34.index
   69108    70798    73587    74230    75132    76210    76584    78408
33.index 24.index 19.thumb
   79968    82244    84715
```

```
rw.7k <- rrarefy(rw.table.tmp, 7000)
rm(streptophyta, mitochondria, rw.table.tmp)
```

Since lots of OTUs were removed from the OTU table, we remove them from the taxonomy table - we will want everything lined up downstream.

```
rw.taxo.7k <- rw.taxo[colnames(rw.7k), ]
```

```
rm(rw.taxo)
```

So we're left with 357000 sequences in 51 samples and 7404 OTUs.

---

### Configure sample metadata

The mapping file (metadata for each sample) was loaded in during the first step. First, we line up samples with the OTU table row names since it is now in shape. Then there is lots of baggage that comes along with mapping files. Factor variables must be retrained, and then we add three colors that will be used in analysis.

```
# mapping file
map <- rw.map[row.names(rw.7k), ]  # reorder to match
rm(rw.map)  # remove old one

# then reorder a few factors for convenience.
map$individ <- factor(map$individ, levels=c(as.character(levels(map$individ)[1:17])))
map$location <- factor(map$location, levels=c('index', 'thumb', 'phone'))
map$type <- factor(map$type, levels=c('c', 'o', 'p'))
```

```
map$dominance <- factor(map$dominance, levels=c('r', 'l'))
map$gender <- factor(map$gender, levels=c('f', 'm'))
map$wash <- factor(map$wash, levels=c('y', 'n'))
map <- map[, c(3, 5, 7, 8, 9)]

# create colors for plotting ease
map$bg <- 'gray30'  # phones
map$bg[map$location == 'index'] <- 'cornflowerblue'
map$bg[map$location == 'thumb'] <- 'darkorange'
```

And create a few more variables.

```
map$loc.gen <- paste(map$location, map$gender, sep='.')
map$location2 <- as.character(map$location)
map$location2[map$location2 != 'phone'] <- 'finger'
map$location2 <- factor(map$location2)
map$loc.gen2 <- factor(paste(map$location2, map$gender, sep='.'),
                       levels=c('finger.f', 'phone.f', 'finger.m', 'phone.m'))

index <- which(map$location == 'index')
thumb <- which(map$location == 'thumb')
finger <- which(map$location2 == 'finger')

m <- which(map$gender == 'm')
f <- which(map$gender == 'f')
p <- which(map$location == 'phone')
```

Also, a quick function to print simple LaTeXtables over and over.

```
pxtable <- function(tab, capt='') {
  print(xtable(tab, caption=capt), comment=FALSE, timestamp=FALSE)#, type='html')
  }
```

### Generate taxonomy figure

Taxonomy information, as QIIME gives it, is pretty useless raw. So we have to parse this into a workable data frame, and then use that for figures. First, rename and save on typing! Then the separation between taxonomic levels is used to split strings. A couple more steps and then we have a data frame with 7 taxonomic levels and one last column for total abundance across the rarefied dataset.

```
tt <- rw.taxo.7k
tt2 <- as.character(gsub('[[:alpha:]]{1,1}\\_\\_', '', tt$taxa.names))
tt3 <- strsplit(tt2, split='; ')
ttl <- unlist(lapply(tt3, length))

tt4 <- data.frame(
  kingdom=sapply(tt3, function(x){x[1]}),
  phylum=sapply(tt3, function(x){x[2]}),
  class=sapply(tt3, function(x){x[3]}),
  order=sapply(tt3, function(x){x[4]}),
  family=sapply(tt3, function(x){x[5]}),
  genus=sapply(tt3, function(x){x[6]}),
```

```
  species=sapply(tt3, function(x){x[7]}))

tt4$kingdom <- as.character(tt4$kingdom)
tt4$phylum <- as.character(tt4$phylum)
tt4$class <- as.character(tt4$class)
tt4$order <- as.character(tt4$order)
tt4$family <- as.character(tt4$family)
tt4$genus <- as.character(tt4$genus)
tt4$species <- as.character(tt4$species)

for (i in 1:ncol(tt4)){
  tt4[which(is.na(tt4[, i])), i] <- ''
    } # warning suppressed

taxo <- tt4
taxo$abundance <- colSums(rw.7k)
row.names(taxo) <- rw.taxo.7k$qiime.id
rm(tt, tt2, tt3, ttl, tt4)

head(taxo)
```

```
   kingdom         phylum               class              order
3  Bacteria Proteobacteria Gammaproteobacteria   Pseudomonadales
11 Bacteria Proteobacteria Gammaproteobacteria Enterobacteriales
16 Bacteria    Tenericutes          Mollicutes Acholeplasmatales
30 Bacteria Planctomycetes      Planctomycetia        Gemmatales
31 Bacteria     Firmicutes          Clostridia     Clostridiales
42 Bacteria    Chloroflexi               C0119
               family         genus    species abundance
3       Moraxellaceae Enhydrobacter aerosaccus         0
11 Enterobacteriaceae                                  1
16 Acholeplasmataceae  Acholeplasma                    1
30     Isosphaeraceae                                  0
31    Veillonellaceae     Dialister                  231
42                                                     1
```

Looks good. Then we want to know about the most abundant phyla, to be used for a taxonomy figure. But first, what was the most abundant thing in the dataset?

```
medAbu5 <- sort(apply(rw.7k, 2, median)/25, TRUE)[1:5]
medAbu5.m <- sort(apply(rw.7k[m, ], 2, median)/70, TRUE)[1:5]
medAbu5.f <- sort(apply(rw.7k[f, ], 2, median)/70, TRUE)[1:5]
medAbu5.p <- sort(apply(rw.7k[p, ], 2, median)/70, TRUE)[1:5]
medAbu5.fing <- sort(apply(rw.7k[finger, ], 2, median)/70, TRUE)[1:5]

pxtable(data.frame(taxo[names(medAbu5), -c(1,3,7,8)], medAbu=medAbu5),
        capt='Most median abundant overall.')
```

```
pxtable(data.frame(taxo[names(medAbu5.m), -c(1,3,7,8)], medAbu=medAbu5.m),
        capt='Most median abundant for men.')
```

| | phylum | order | family | genus | medAbu |
|---|---|---|---|---|---|
| 8696 | Firmicutes | Lactobacillales | Streptococcaceae | Streptococcus | 26.36 |
| 30072 | Firmicutes | Bacillales | Staphylococcaceae | Staphylococcus | 23.04 |
| 27600 | Actinobacteria | Actinomycetales | Corynebacteriaceae | Corynebacterium | 7.68 |
| 20343 | Firmicutes | Lactobacillales | Streptococcaceae | Streptococcus | 7.32 |
| 14703 | Proteobacteria | Pasteurellales | Pasteurellaceae | Haemophilus | 7.08 |

Table 1: Most median abundant overall.

| | phylum | order | family | genus | medAbu |
|---|---|---|---|---|---|
| 30072 | Firmicutes | Bacillales | Staphylococcaceae | Staphylococcus | 9.36 |
| 27600 | Actinobacteria | Actinomycetales | Corynebacteriaceae | Corynebacterium | 6.21 |
| 8696 | Firmicutes | Lactobacillales | Streptococcaceae | Streptococcus | 6.03 |
| 14703 | Proteobacteria | Pasteurellales | Pasteurellaceae | Haemophilus | 1.36 |
| 23224 | Actinobacteria | Actinomycetales | Corynebacteriaceae | Corynebacterium | 1.33 |

Table 2: Most median abundant for men.

```
pxtable(data.frame(taxo[names(medAbu5.f), -c(1,3,7,8)], medAbu=medAbu5.f),
        capt='Most median abundant for women.')
```

| | phylum | order | family | genus | medAbu |
|---|---|---|---|---|---|
| 8696 | Firmicutes | Lactobacillales | Streptococcaceae | Streptococcus | 13.49 |
| 30072 | Firmicutes | Bacillales | Staphylococcaceae | Staphylococcus | 7.09 |
| 20343 | Firmicutes | Lactobacillales | Streptococcaceae | Streptococcus | 5.13 |
| 14703 | Proteobacteria | Pasteurellales | Pasteurellaceae | Haemophilus | 3.26 |
| 30140 | Firmicutes | Clostridiales | Veillonellaceae | Veillonella | 1.46 |

Table 3: Most median abundant for women.

```
pxtable(data.frame(taxo[names(medAbu5.p), -c(1,3,7,8)], medAbu=medAbu5.p),
        capt='Most median abundant on phones.')
```

```
pxtable(data.frame(taxo[names(medAbu5.fing), -c(1,3,7,8)], medAbu=medAbu5.fing),
        capt='Most median abundant on fingers.')
```

| | phylum | order | family | genus | medAbu |
|---|---|---|---|---|---|
| 8696 | Firmicutes | Lactobacillales | Streptococcaceae | Streptococcus | 11.81 |
| 30072 | Firmicutes | Bacillales | Staphylococcaceae | Staphylococcus | 6.19 |
| 27600 | Actinobacteria | Actinomycetales | Corynebacteriaceae | Corynebacterium | 4.26 |
| 14703 | Proteobacteria | Pasteurellales | Pasteurellaceae | Haemophilus | 3.33 |
| 20343 | Firmicutes | Lactobacillales | Streptococcaceae | Streptococcus | 2.80 |

Table 4: Most median abundant on phones.

| | phylum | order | family | genus | medAbu |
|---|---|---|---|---|---|
| 30072 | Firmicutes | Bacillales | Staphylococcaceae | Staphylococcus | 10.10 |
| 8696 | Firmicutes | Lactobacillales | Streptococcaceae | Streptococcus | 9.24 |
| 20343 | Firmicutes | Lactobacillales | Streptococcaceae | Streptococcus | 2.61 |
| 14703 | Proteobacteria | Pasteurellales | Pasteurellaceae | Haemophilus | 2.43 |
| 27600 | Actinobacteria | Actinomycetales | Corynebacteriaceae | Corynebacterium | 1.78 |

Table 5: Most median abundant on fingers.

For all groups, it is either a *Staphylococcus* or *Streptococcus* OTU. The median abundance was used because OTU abundances can be easily skewed by outlier values (as is also the case in this dataset).

```
ph <- aggregate(taxo$abundance, by=list(taxo$phylum), FUN=sum)
ph[rev(order(ph$x))[1:10], ]
```

```
          Group.1      x
15     Firmicutes 175110
4  Actinobacteria  82129
21 Proteobacteria  65027
1                  13198
6   Bacteroidetes  12922
16   Fusobacteria   3805
25    Tenericutes   3462
11  Cyanobacteria    317
2        [Thermi]    219
3   Acidobacteria    203
```

```
#  cut off after fusobacteria and group into 'other'
```

We can use the top 5 and group all others. So a new data frame is created to hold the mean abundances grouped by phylum and by location type (index, thumb, or phone). This code is not pretty but it works.

```
ph.mean <- data.frame(
  Firmicutes = aggregate(rowSums(rw.7k[, which(taxo$phylum == 'Firmicutes')]),
    by=list(map$location), FUN=mean),
  Actinobacteria = aggregate(rowSums(rw.7k[, which(taxo$phylum == 'Actinobacteria')]),
    by=list(map$location), FUN=mean),
    Proteobacteria = aggregate(rowSums(rw.7k[, which(taxo$phylum == 'Proteobacteria')]),
    by=list(map$location), FUN=mean),
  Bacteroidetes = aggregate(rowSums(rw.7k[, which(taxo$phylum == 'Bacteroidetes')]),
    by=list(map$location), FUN=mean),
    Fusobacteria = aggregate(rowSums(rw.7k[, which(taxo$phylum == 'Fusobacteria')]),
    by=list(map$location), FUN=mean),
    Other = aggregate(rowSums(rw.7k[, -c(which(taxo$phylum %in%
        c('Firmicutes', 'Actinobacteria', 'Proteobacteria',
      'Bacteroidetes', 'Fusobacteria')))]),
        by=list(map$location), FUN=mean))

ph.mean <- ph.mean[, c(2, 4, 6, 8, 10, 12)]
row.names(ph.mean) <- c('index', 'thumb', 'phone')
names(ph.mean) <- gsub('.x', '', names(ph.mean))
ph.mean <- ph.mean/7000
```

Then the same thing is done, but to generate standard errors for bar graph error bars. It is the same big ugly code chunk, but FUN=sd is used as the final arguement. SE must be calculated by hand in r, so there is one extra step, and then they are reversed so the big bars are on top in descending order.

```
se <- function(x) {sd(x)/sqrt(length(x))}
rw.7k.rel <- rw.7k/7000

ph.se <- data.frame(
```

```
    Firmicutes = aggregate(rowSums(rw.7k.rel[, which(taxo$phylum == 'Firmicutes')]),
    by=list(map$location), FUN=se),
    Actinobacteria = aggregate(rowSums(rw.7k.rel[, which(taxo$phylum == 'Actinobacteria')]),
    by=list(map$location), FUN=se),
  Proteobacteria = aggregate(rowSums(rw.7k.rel[, which(taxo$phylum == 'Proteobacteria')]),
    by=list(map$location), FUN=se),
    Bacteroidetes = aggregate(rowSums(rw.7k.rel[, which(taxo$phylum == 'Bacteroidetes')]),
    by=list(map$location), FUN=se),
  Fusobacteria = aggregate(rowSums(rw.7k.rel[, which(taxo$phylum == 'Fusobacteria')]),
    by=list(map$location), FUN=se),
    Other = aggregate(rowSums(rw.7k.rel[, -c(which(taxo$phylum %in%
        c('Firmicutes', 'Actinobacteria', 'Proteobacteria',
      'Bacteroidetes', 'Fusobacteria')))]),
        by=list(map$location), FUN=se))
ph.se <- ph.se[, c(2, 4, 6, 8, 10, 12)]
row.names(ph.se) <- c('index', 'thumb', 'phone')
names(ph.se) <- gsub('.x', '', names(ph.se))
```

And then turn them upside down for nicer plotting.

```
ph.mean <- ph.mean[, c(6:1)]
ph.se <- ph.se[, c(6:1)]
```

Now we have data in place to make a barplot by hand:

```
par(mar=c(5,7,2,2), las=1, font.lab=1)
mids <- barplot(as.matrix(ph.mean), beside=TRUE, horiz=TRUE, las=1, xlim=c(0,.6),
  border='white', xlab='', axisnames=FALSE,
  col=c('cornflowerblue', 'darkorange', 'gray30'), xaxt='n', font.lab=2)
abline(v=c(seq(.1, .6, .1)), col='white', lwd=.5)
arrows(unlist(c(ph.mean-ph.se)), unlist(c(mids)),
       unlist(c(ph.mean+ph.se)), unlist(c(mids)),
    code=3, angle=90, length=.01)
axis(1, at=c(0,.1,.2,.3,.4,.5,.6), labels=c(0,10,20,30,40,50,60))
legend(.40, 7, legend=c('phone', 'thumb', 'index'), pch=15, pt.cex=2,
    col=c('gray30', 'darkorange', 'cornflowerblue'), bty='n', y.intersp=.82)
mtext('Percent of Each Sample', side=1, line=2.4, font=2)
mtext(names(ph.mean), side=2, at=c(mids[2, ]), line=.2, font=3)
```

Now try to hone in on the Firmicutes since that is the most prominent difference:

*Note - One OTU (Paenibacillus) appears to be an outlier with a wacky distribution, so it is left out. Although* `set.seed` *is used at the top, it might show up in the top 10 again if it is all run again with different parameters.*

```
#Firmicutes = aggregate(rowSums(rw.7k.rel[, which(taxo$phylum == 'Firmicutes')]),
#    by=list(map$location), FUN=se),
fir.table <- rw.7k.rel[, which(taxo$phylum == 'Firmicutes')]
fir.taxo <- taxo[taxo$phylum == 'Firmicutes', ]
identical(colnames(fir.table), row.names(fir.taxo))
```

```
[1] TRUE
```

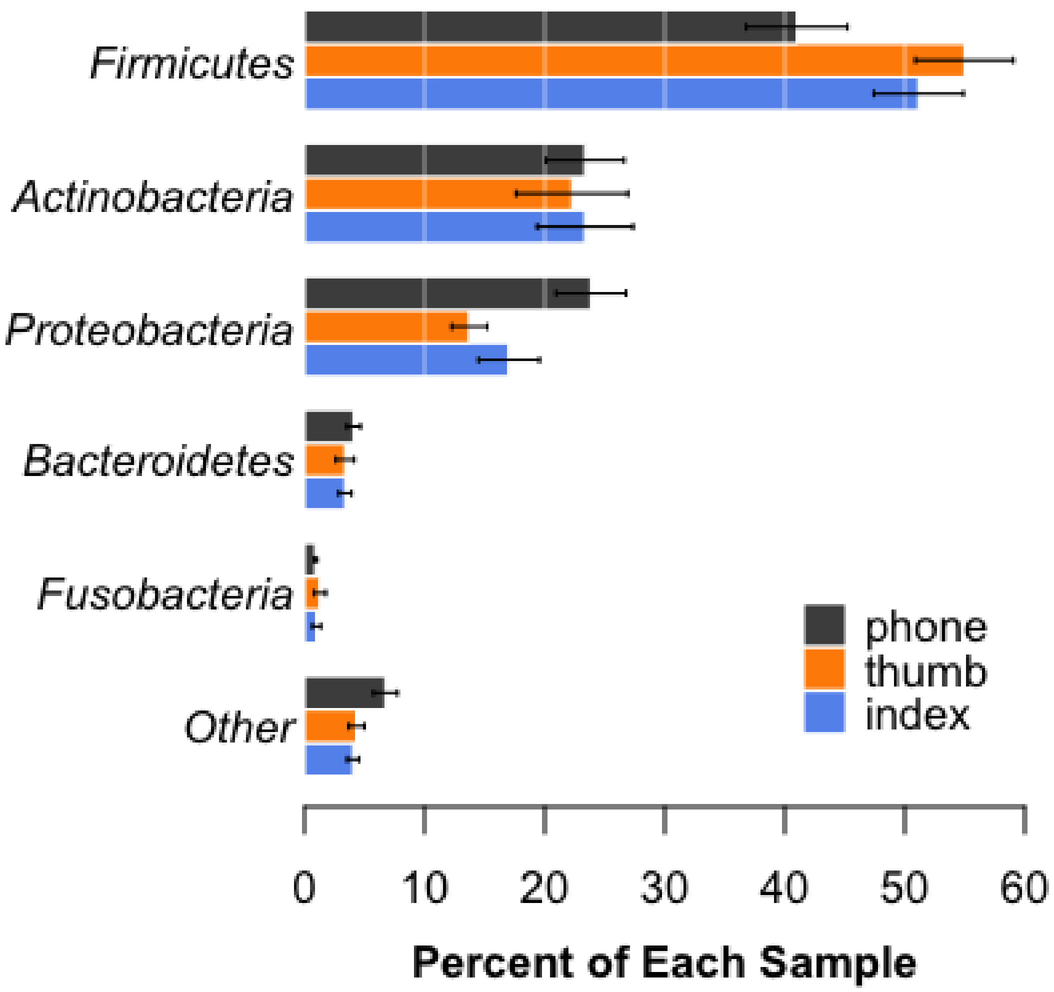


Figure 1: plot of chunk phylumBarplot

```
# leave out outlier
fir.table.10 <- fir.table[, names(rev(sort(colSums(fir.table))))[c(1:4, 6:11)]]  #
fir.taxo.10 <- fir.taxo[colnames(fir.table.10), ]
dim(fir.table.10)
```

```
[1] 51 10
```

```
sum(colMeans(fir.table.10))
```

```
[1] 0.3915
```

What percentage of observations are represented here? 39.1541%

*Note, again, that one of the original top 10 shows an ugly distribution = all driven by one person with A LOT of Paenibacillus on his fingers. So that one was removed in the code above for clarity - now we're looking at `c(1:4, 6:11)` to make an even 10. For further investigation, that OTU was ID# 29684. Oddly, it was elevated on both fingers, but didn't show up on his phone. It was nearly absent from all other samples.*

Now create data frames to hold plotting info.

```
fir.mean <- data.frame(matrix(NA, 4, 10))
row.names(fir.mean) <- levels(map$loc.gen2)
names(fir.mean) <- colnames(fir.table.10)

fir.se <- data.frame(matrix(NA, 4, 10))
row.names(fir.se) <- levels(map$loc.gen2)
names(fir.se) <- colnames(fir.table.10)

test <- aggregate(fir.table.10[, i], by=list(map$loc.gen2), mean)

if(identical(row.names(fir.mean), as.character(test$Group.1))) {
  for (i in 1:10) {
    fir.mean[, i] <- aggregate(fir.table.10[, i], by=list(map$loc.gen2), mean)$x
    fir.se[, i] <- aggregate(fir.table.10[, i], by=list(map$loc.gen2), se)$x
    }
} else {print("Didn't work - rows weren't lined up!")}

fir.mean <- fir.mean[4:1, 10:1] * 100
fir.se <- fir.se[4:1, 10:1] * 100
```

And do the same for Actinobacteria.

```
act.table <- rw.7k.rel[, which(taxo$phylum == 'Actinobacteria')]
act.taxo <- taxo[taxo$phylum == 'Actinobacteria', ]
identical(colnames(act.table), row.names(act.taxo))
```

```
[1] TRUE
```

```
act.table.10 <- act.table[, names(rev(sort(colSums(act.table))))[c(1:10)]]
act.taxo.10 <- act.taxo[colnames(act.table.10), ]
dim(act.table.10)
```

```
[1] 51 10
```

```r
sum(colMeans(act.table.10))
```

```
[1] 0.1743
```

What percentage of observations are represented here? 17.4325%

```r
act.mean <- data.frame(matrix(NA, 4, 10))
row.names(act.mean) <- levels(map$loc.gen2)
names(act.mean) <- colnames(act.table.10)

act.se <- data.frame(matrix(NA, 4, 10))
row.names(act.se) <- levels(map$loc.gen2)
names(act.se) <- colnames(act.table.10)

test <- aggregate(act.table.10[, i], by=list(map$loc.gen2), mean)

if(identical(row.names(act.mean), as.character(test$Group.1))) {
  for (i in 1:10) {
    act.mean[, i] <- aggregate(act.table.10[, i], by=list(map$loc.gen2), mean)$x
    act.se[, i] <- aggregate(act.table.10[, i], by=list(map$loc.gen2), se)$x
    }
} else {print("Didn't work - rows weren't lined up!")}

act.mean <- act.mean[4:1, 10:1] * 100
act.se <- act.se[4:1, 10:1] * 100
```

And do the same for Proteobacteria.

```r
pro.table <- rw.7k.rel[, which(taxo$phylum == 'Proteobacteria')]
pro.taxo <- taxo[taxo$phylum == 'Proteobacteria', ]
identical(colnames(pro.table), row.names(pro.taxo))
```

```
[1] TRUE
```

```r
pro.table.10 <- pro.table[, names(rev(sort(colSums(pro.table)))[c(1:10)])]
pro.taxo.10 <- fir.taxo[colnames(pro.table.10), ]
dim(pro.table.10)
```

```
[1] 51 10
```

```r
sum(colMeans(pro.table.10))
```

```
[1] 0.1109
```

What percentage of observations are represented here? 11.0888%

```r
pro.mean <- data.frame(matrix(NA, 4, 10))
row.names(pro.mean) <- levels(map$loc.gen2)
names(pro.mean) <- colnames(pro.table.10)

pro.se <- data.frame(matrix(NA, 4, 10))
row.names(pro.se) <- levels(map$loc.gen2)
names(pro.se) <- colnames(pro.table.10)

test <- aggregate(pro.table.10[, i], by=list(map$loc.gen2), mean)

if(identical(row.names(pro.mean), as.character(test$Group.1))) {
  for (i in 1:10) {
    pro.mean[, i] <- aggregate(pro.table.10[, i], by=list(map$loc.gen2), mean)$x
    pro.se[, i] <- aggregate(pro.table.10[, i], by=list(map$loc.gen2), se)$x
    }
} else {print("Didn't work - rows weren't lined up!")}

pro.mean <- pro.mean[4:1, 10:1] * 100
pro.se <- pro.se[4:1, 10:1] * 100
```

So now try to combine them. One error bar spills of the right margin, but it is not worth throwing off the whole balance of the figure. So instead I wrote a simple break at the margin to report the real value. *Might move around if re-rarefied without setting seed. So it goes.*

```r
# pdf('phylumBarplot.pdf', height=6, width=15, useDingbats=FALSE)
# postscript('phylumBarplot.eps', height=5, width=15)

name.cex <- 0.9

# par(mfrow=c(1,2))
layout(matrix(c(1,2,3,4), 1, 4), widths=c(1, 1, 1, 1))
par(mar=c(5,9,2,2), las=1, font.lab=1,
    fg='gray20', col.axis='gray20', col.lab='gray20')
mids <- barplot(as.matrix(ph.mean), beside=TRUE, horiz=TRUE, las=1, xlim=c(0,.6),
  border='white', xlab='', axisnames=FALSE,
  col=c('cornflowerblue', 'darkorange', 'gray30'), xaxt='n', font.lab=2)
abline(v=c(seq(.1, .6, .1)), col='white', lwd=.5)
arrows(unlist(c(ph.mean-ph.se)), unlist(c(mids)),
     unlist(c(ph.mean+ph.se)), unlist(c(mids)),
    code=3, angle=90, length=.01)
axis(1, at=c(0,.1,.2,.3,.4,.5,.6), labels=c(0,10,20,30,40,50,60))
legend(.40, 4, legend=c('phone', 'thumb', 'index'), pch=15, pt.cex=3, cex=1.4,
    col=c('gray30', 'darkorange', 'cornflowerblue'), bty='n', y.intersp=.9)
mtext('Percent of Each Sample', side=1, line=2.4, font=2)
mtext('(a) Most abundant phyla', side=3, line=0, font=2, at=0, adj=0)
mtext(names(ph.mean), side=2, at=c(mids[2, ]), line=.2, font=1)
par(xpd=TRUE)
segments(0, c(mids[1, ]-.45), 0, c(mids[3, ]+.45))
par(xpd=FALSE)

# Firmicutes
par(mar=c(5,8,2,2), las=1, font.lab=1, xpd=FALSE,
    fg='gray20', col.axis='gray20', col.lab='gray20')
```

```
mids <- barplot(as.matrix(fir.mean), beside=TRUE, horiz=TRUE, las=1, xlim=c(0,30),
  border='white', axisnames=FALSE,
  col=c('gray30', 'cornflowerblue'), font.lab=2)
abline(v=c(seq(1, 30, 5)), col='white', lwd=.5)
arrows(unlist(c(fir.mean-fir.se)), unlist(c(mids)),
     unlist(c(fir.mean+fir.se)), unlist(c(mids)),
  code=3, angle=90, length=.01)
legend(15, mids[5], legend=c('phone', 'index'), pch=15, pt.cex=3, cex=1.4,
  col=c('gray30', 'cornflowerblue'), bty='n', y.intersp=.9)
mtext('Percent of Each Sample', side=1, line=2.4, font=2)
mtext('(b) Firmicutes', side=3, line=0, font=2, at=0, adj=0)

par(xpd=TRUE)
segments(0, c(mids[1, ]-.45), 0, c(mids[4, ]+.45))
for (i in 1:10) {
  segments(-.05, mean(mids[, i]), -1.7, mean(mids[, i]), col='gray60')
  text(-1, mean(mids[1:2, i]),
     "\\MA",vfont=c("sans serif symbol","plain"), font=2, col='gray20', cex=1.5)
  text(-1, mean(mids[3:4, i]),
     "\\VE",vfont=c("sans serif symbol","plain"), font=2, col='gray20', cex=1.5)
  barname <- taxo[names(fir.mean)[i], 'genus']
  font <- 3
  if (barname == '') {
    barname <- taxo[names(fir.mean)[i], 'family']
    font <- 1}
  mtext(barname, side=2, at=mean(mids[, i]), line=1.5, font=font, cex=name.cex)
}

# Actinobacteria
par(mar=c(5,8,2,2), las=1, font.lab=1, xpd=FALSE,
    fg='gray20', col.axis='gray20', col.lab='gray20')
mids <- barplot(as.matrix(act.mean), beside=TRUE, horiz=TRUE, las=1, xlim=c(0,15),
  border='white', axisnames=FALSE,
  col=c('gray30', 'cornflowerblue'), font.lab=2)
abline(v=c(seq(1, 8, 1)), col='white', lwd=.5)
arrows(unlist(c(act.mean-act.se)), unlist(c(mids)),
     unlist(c(act.mean+act.se)), unlist(c(mids)),
  code=3, angle=90, length=.01)
mtext('Percent of Each Sample', side=1, line=2.4, font=2)
mtext('(c) Actinobacteria', side=3, line=0, font=2, at=0, adj=0)

par(xpd=TRUE)
segments(0, c(mids[1, ]-.45), 0, c(mids[4, ]+.45))
for (i in 1:10) {
  segments(-.05, mean(mids[, i]), -.8, mean(mids[, i]), col='gray60')
  text(-.45, mean(mids[1:2, i]),
     "\\MA",vfont=c("sans serif symbol","plain"), font=2, col='gray20', cex=1.5)
  text(-.45, mean(mids[3:4, i]),
     "\\VE",vfont=c("sans serif symbol","plain"), font=2, col='gray20', cex=1.5)
  barname <- taxo[names(act.mean)[i], 'genus']
  font <- 3
  if (barname == '') {
    barname <- taxo[names(act.mean)[i], 'family']
```

```
    font <- 1}
  mtext(barname, side=2, at=mean(mids[, i]), line=1.5, font=font, cex=name.cex)
}

# Proteobacteria
par(mar=c(5,8,2,2.5), las=1, font.lab=1, xpd=FALSE,
    fg='gray20', col.axis='gray20', col.lab='gray20')
mids <- barplot(as.matrix(pro.mean), beside=TRUE, horiz=TRUE, las=1, xlim=c(0,8),
  border='white', axisnames=FALSE,
  col=c('gray30', 'cornflowerblue'), font.lab=2)
abline(v=c(seq(1, 8, 1)), col='white', lwd=.5)
arrows(unlist(c(pro.mean-pro.se)), unlist(c(mids)),
     unlist(c(pro.mean+pro.se)), unlist(c(mids)),
  code=3, angle=90, length=.01)
mtext('Percent of Each Sample', side=1, line=2.4, font=2)
mtext('(d) Proteobacteria', side=3, line=0, font=2, at=0, adj=0)

par(xpd=TRUE)
segments(0, c(mids[1, ]-.45), 0, c(mids[4, ]+.45))
for (i in 1:10) {
  segments(-.05, mean(mids[, i]), -.5, mean(mids[, i]), col='gray60')
  text(-.25, mean(mids[1:2, i]),
     "\\MA",vfont=c("sans serif symbol","plain"), font=2, col='gray20', cex=1.5)
  text(-.25, mean(mids[3:4, i]),
     "\\VE",vfont=c("sans serif symbol","plain"), font=2, col='gray20', cex=1.5)
  barname <- taxo[names(pro.mean)[i], 'genus']
  font <- 3
  if (barname == '') {
    barname <- taxo[names(pro.mean)[i], 'family']
    font <- 1 }
  mtext(barname, side=2, at=mean(mids[, i]), line=1.5, font=font, cex=name.cex)
}
```

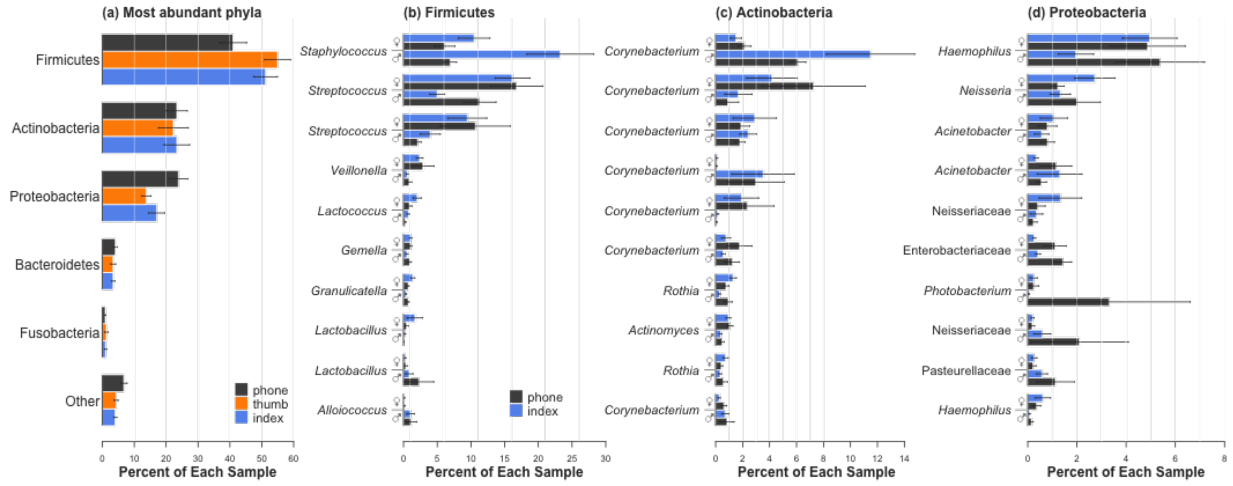


Figure 2: plot of chunk longPhylumBarplots

```r
# dev.off()
```

---

**Jaccard Distance Barplots - how are phones related to people?**

Next, we want to know how communities break out between people and their phones. To do this, we make a distance matrix. In our case, we want to be able to easily explain so we use Jaccard similarity

$$S_{jaccard} = \frac{shared\ richness}{combined\ richness}$$

so that we can interpret in easy language. Later, we'll also want a *similarity* rather than a *distance*, so we'll invert the distance R gives by default

$$S_{jaccard} = 1 - D_{jaccard}$$

. This way things with more in common have higher values, and that is easier to visualize.

Note that this was tried also with the same Canberra distance that will be used later for ordinations. Results were almost identical, but Jaccard is much easier to interpret for this sort of graph, so we use Jaccard. Also, the Jaccard calculation in the `vegan` package is not the one we want, so I've written one that calculates the original Jaccard similarity. This takes a bit of time since it is not compiled. Thus, this is chached so that it does not have to run every time. If rerunning with slightly different data, unchache this code chunk. So it goes.

```r
JacJFM <- function(x=rw.7k) {
  mat <- matrix(0, nrow(x), nrow(x))
  mat.names <- dimnames(rw.7k)[[1]]
  dimnames(mat) <- list(mat.names, mat.names)

  for(i in 1:nrow(mat)) {
    for(j in 1:ncol(mat)) {
      iname <- row.names(x)[i]
      jname <- row.names(x)[j]
      if(iname != jname) {
        ijcount <- apply(x[c(iname, jname), ]>1, 2, sum)
        ijintersect <- length(which(ijcount == 2))
        ijunion <- length(which(ijcount > 0))
        ijvalue <- ijintersect/ijunion
        mat[iname, jname] <- ijvalue
      }
    }
  }
  invisible(as.dist(mat))
}

dis <- 1-JacJFM()
```

Since we want to do this several times, I'll package a few tedious routines into functions to cut down on repetative coding. First set up a data frame for the whole dataset.

```r
bar.df <- data.frame(matrix(0,17,3))
names(bar.df) <- c('in.th', 'in.ph', 'th.ph')
row.names(bar.df) <- unique(map$individ)
```

Then create the functions that will be used a few times.

```
makeBarDF <- function() {
  for(i in 1:nrow(bar.df)) {
  bar.df[i, 1] <- as.matrix(dis)[which(map$individ == row.names(bar.df)[i] &
                                         map$location == 'index'),
                                   which(map$individ == row.names(bar.df)[i] &
                                         map$location == 'thumb')]
    bar.df[i, 2] <- as.matrix(dis)[which(map$individ == row.names(bar.df)[i] &
                                         map$location == 'index'),
                                   which(map$individ == row.names(bar.df)[i] &
                                         map$location == 'phone')]
    bar.df[i, 3] <- as.matrix(dis)[which(map$individ == row.names(bar.df)[i] &
                                         map$location == 'thumb'),
                                   which(map$individ == row.names(bar.df)[i] &
                                         map$location == 'phone')]
  }
  invisible(bar.df)
}

makeBarSummary <- function(bar.df=bar.df) {
  bar.summary <- data.frame(cbind(apply(bar.df, 2, mean),
                              (apply(bar.df, 2, sd)/sqrt(nrow(bar.df)))))
  bar.summary[, 3] <- bar.summary[, 1]-bar.summary[, 2]
  bar.summary[, 4] <- bar.summary[, 1]+bar.summary[, 2]
  names(bar.summary) <- c('mean', 'se', 'se.lo', 'se.hi')
  invisible(bar.summary)
}
```

And then run the data set through the functions.

```
bar.jac.df <- makeBarDF()
bar.jac <- makeBarSummary(bar.df=bar.jac.df)

bar.jac.df
```

```
    in.th  in.ph  th.ph
17 0.6521 0.7753 0.7842
18 0.6566 0.8237 0.7965
19 0.6992 0.7612 0.8006
20 0.7080 0.8396 0.8559
22 0.6481 0.7939 0.8152
23 0.6284 0.6860 0.7461
24 0.6772 0.8354 0.8132
25 0.6656 0.7896 0.7964
26 0.7507 0.8030 0.8162
28 0.8034 0.7923 0.8459
29 0.6745 0.7487 0.7550
30 0.7104 0.8421 0.8132
31 0.6959 0.8483 0.8398
32 0.7473 0.7214 0.7023
33 0.6420 0.6961 0.6511
34 0.5786 0.7769 0.7842
35 0.7698 0.7959 0.7746
```

```
bar.jac
```

```
        mean      se  se.lo  se.hi
in.th 0.6887 0.01367 0.6750 0.7024
in.ph 0.7841 0.01192 0.7722 0.7960
th.ph 0.7877 0.01256 0.7751 0.8002
```

Each additional time, we're only interested in a few samples at a time, so run subsets through the same functions. Each starts out being named generically `bar.df`, but then each object gets put into a uniquely named data frame.

First we need to know how many are in each group.

```
table(map$gender)/3
```

```
 f  m 
10  7 
```

```
table(map$wash)/3
```

```
y n 
9 8 
```

```
# females=10
bar.df <- data.frame(matrix(0,10,3))
names(bar.df) <- c('in.th', 'in.ph', 'th.ph')
row.names(bar.df) <- unique(map$individ[which(map$gender == 'f')])
bar.df.female.j <- makeBarDF()
bar.female.j <- makeBarSummary(bar.df=bar.df.female.j)

# males=7
bar.df <- data.frame(matrix(0,7,3))
names(bar.df) <- c('in.th', 'in.ph', 'th.ph')
row.names(bar.df) <- unique(map$individ[which(map$gender == 'm')])
bar.df.male.j <- makeBarDF()
bar.male.j <- makeBarSummary(bar.df=bar.df.male.j)

# yes wash=9
bar.df <- data.frame(matrix(0,9,3))
names(bar.df) <- c('in.th', 'in.ph', 'th.ph')
row.names(bar.df) <- unique(map$individ[which(map$wash == 'y')])
bar.df.wash.j <- makeBarDF()
bar.wash.j <- makeBarSummary(bar.df=bar.df.wash.j)

# no wash=8
bar.df <- data.frame(matrix(0,8,3))
names(bar.df) <- c('in.th', 'in.ph', 'th.ph')
row.names(bar.df) <- unique(map$individ[which(map$wash == 'n')])
bar.df.nowash.j <- makeBarDF()
bar.nowash.j <- makeBarSummary(bar.df=bar.df.nowash.j)
```

To make it easeier to combine side by side barplots, we'll combine them into joined data frames based on their variables (males and females, and wash and no-wash). Then one last step to invert the numbers from a distance to a similarity.

```
bar.mf <- data.frame(rbind(bar.male.j[1, ], bar.female.j[1, ],
                       bar.male.j[2, ], bar.female.j[2, ],
                           bar.male.j[3, ], bar.female.j[3, ]))
bar.wnw <- data.frame(rbind(bar.wash.j[1, ], bar.nowash.j[1, ],
                           bar.wash.j[2, ], bar.nowash.j[2, ],
                           bar.wash.j[3, ], bar.nowash.j[3, ]))

bar.jac <- 1-bar.jac
bar.mf <- 1-bar.mf
bar.wnw <- 1-bar.wnw
```

All data are in place, so there is lots of futzy code to get barplots to look nice. These were modeled after Edward Tufte's *The Visual Display of Quantitative Information.*

```
# pdf('longBarplotFigure.pdf', width=8, height=4)
# postscript('longBarplotFigure.eps', width=8, height=4)

ylim <- c(0,.45)
layout(matrix(c(1,2,3), 1,3), widths=c(1, 1.6, 1.6))
par(mar=c(4, 4.5, 2, 1), las=1, fg='gray20', lheight=1, col.axis='gray20', col.lab='gray20')
mids <- barplot(bar.jac$mean, las=1,
  border='transparent', axes=FALSE, ylim=ylim, yaxs='i',
    # ylab='Percent of species in common')
  ylab='')
mtext('Jaccard Similarity\n(as % of shared OTUs)', side=2, line=2, las=0, cex=.8)
abline(h=c(.1,.2,.3,.4), col='white', lwd=1)
mtext(c('index\n&\nthumb', 'index\n&\nphone', 'thumb\n&\nphone'),
    side=1, line=2.1, at=c(mids), cex=.7, col='gray20')
axis(2, col='gray20', col.ticks='gray20',
    at=c(0,.1,.2,.3,.4), labels=c(0,10,20,30,'40%'))
arrows(mids, bar.jac$se.lo, mids, bar.jac$se.hi, code=3,
    angle=90, length=.05, col='gray40')
mtext('(a) All samples', font=2, col='gray20', line=.5)
#
par(mar=c(4, 2, 2, 1))
mids <- barplot(bar.wnw$mean, las=1, col=c('gray80', 'gray50'),
    border='transparent', axes=FALSE, ylim=ylim, yaxs='i')
abline(h=c(.1,.2,.3,.4), col='white', lwd=1)
mtext(c('index\n&\nthumb', 'index\n&\nphone', 'thumb\n&\nphone'),
    side=1, line=2.1, cex=.7, col='gray20',
    at=c(mean(mids[1:2,1]), mean(mids[3:4,1]), mean(mids[5:6,1])))
arrows(mids, bar.wnw$se.lo, mids, bar.wnw$se.hi, code=3,
    angle=90, length=.05, col='gray20')
mtext('(b) Did you wash your hands?', font=2, col='gray20', line=.5)
legend(5, .4, legend=c('yes', 'no'), pch=15, col=c('gray80', 'gray50'),
    pt.cex=2, bty='n')
par(xpd=TRUE)
segments(c(mids[c(1,3,5), 1])-.48, rep(-.00, 3),
         c(mids[c(2,4,6), 1])+.48, rep(-.00, 3),
         col='gray30')
```

```
par(xpd=FALSE)
#
par(mar=c(4, 2, 2, 1))
mids <- barplot(bar.mf$mean, las=1, col=c('gray80', 'gray50'),
  border='transparent', axes=FALSE, ylim=ylim, yaxs='i')
abline(h=c(.1,.2,.3,.4), col='white', lwd=1)
mtext(c('index\n&\nthumb', 'index\n&\nphone', 'thumb\n&\nphone'),
    side=1, line=2.1, cex=.7, col='gray20',
    at=c(mean(mids[1:2,1]), mean(mids[3:4,1]), mean(mids[5:6,1])))
arrows(mids, bar.mf$se.lo, mids, bar.mf$se.hi, code=3,
    angle=90, length=.05, col='gray20')
mtext('(c) Male or Female?', font=2, col='gray20', line=.5)
legend(5, .4, legend=c('m', 'f'), pch=15, col=c('gray80', 'gray50'),
    pt.cex=2, bty='n')
par(xpd=TRUE)
segments(c(mids[c(1,3,5), 1])-.48, rep(-.00, 3),
         c(mids[c(2,4,6), 1])+.48, rep(-.00, 3),
         col='gray30')
```

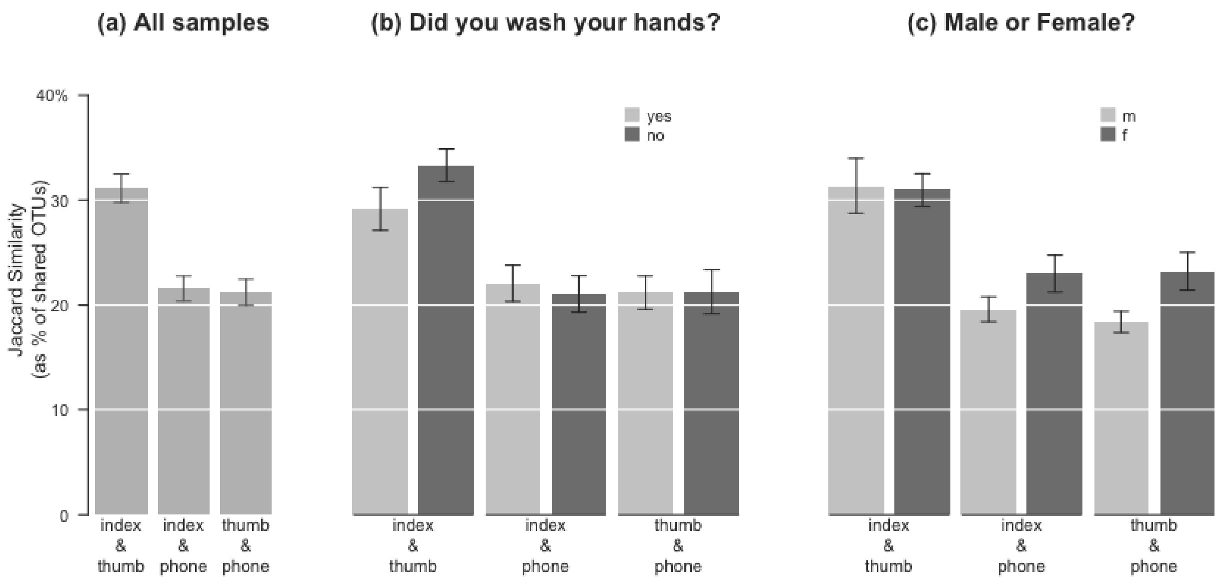


Figure 3: plot of chunk longBarplotFigure

```
par(xpd=FALSE)
#
# dev.off()
```

Isn't that nice? So this figure tells us a couple of really interesting things about the world that we didn't know before! For instance, about 32% of the bacterial taxa we find on our own index finger are also found on the opposing thumb. And (even though fewer) about 22% are also found on our phones! That general pattern is repeated regardless of whether we are looking at men or women, but interestingly, women seem to have more taxa in common with their phones. And your two fingers have more in common if you did not wash your hands.

We can use simple paired t-tests to check some of the patterns in the plots. For instance: Is the first difference significant (are fingers closer to one another than either finger compared to phones)? First, we again need to

invert distances to similarities for easier interpretation (already did this in summary tables, but now for raw distance data frames).

```
bar.jac.df <- 1-bar.jac.df
bar.df.male.j <- 1-bar.df.male.j
bar.df.female.j <- 1-bar.df.female.j
bar.df.wash.j <- 1-bar.df.wash.j
bar.df.nowash.j <- 1-bar.df.nowash.j
```

```
t.test(bar.jac.df$in.th, bar.jac.df$in.ph, paired=TRUE)
```

```
    Paired t-test

data:  bar.jac.df$in.th and bar.jac.df$in.ph
t = 6.075, df = 16, p-value = 0.00001607
alternative hypothesis: true difference in means is not equal to 0
95 percent confidence interval:
 0.0621 0.1287
sample estimates:
mean of the differences
                0.09539
```

```
t.test(bar.jac.df$in.th, bar.jac.df$th.ph, paired=TRUE)
```

```
    Paired t-test

data:  bar.jac.df$in.th and bar.jac.df$th.ph
t = 6.248, df = 16, p-value = 0.00001164
alternative hypothesis: true difference in means is not equal to 0
95 percent confidence interval:
 0.0654 0.1326
sample estimates:
mean of the differences
                0.09898
```

Yes. Very much so. It looks like our fingers have about 32% of their taxa in common, while fingers and phones only share about 22% of taxa.

And how about males and females differentially related to their phones?

```
t.test(bar.df.male.j$in.ph, bar.df.female.j$in.ph)
```

```
    Welch Two Sample t-test

data:  bar.df.male.j$in.ph and bar.df.female.j$in.ph
t = -1.614, df = 14.54, p-value = 0.128
alternative hypothesis: true difference in means is not equal to 0
95 percent confidence interval:
 -0.07966  0.01112
```

```
sample estimates:
mean of x mean of y
   0.1958    0.2300
```

```r
t.test(bar.df.male.j$th.ph, bar.df.female.j$th.ph)
```

```
    Welch Two Sample t-test

data:  bar.df.male.j$th.ph and bar.df.female.j$th.ph
t = -2.336, df = 13.42, p-value = 0.03563
alternative hypothesis: true difference in means is not equal to 0
95 percent confidence interval:
 -0.092371 -0.003746
sample estimates:
mean of x mean of y
   0.1841    0.2321
```

Suggestive but not very strong. And are our fingers more similar if we don't wash our hands?

```r
t.test(bar.df.wash.j$in.th, bar.df.nowash.j$in.th)
```

```
    Welch Two Sample t-test

data:  bar.df.wash.j$in.th and bar.df.nowash.j$in.th
t = -1.625, df = 14.38, p-value = 0.126
alternative hypothesis: true difference in means is not equal to 0
95 percent confidence interval:
 -0.09649  0.01320
sample estimates:
mean of x mean of y
   0.2917    0.3334
```

```r
t.test(bar.df.wash.j$in.ph, bar.df.nowash.j$in.ph)
```

```
    Welch Two Sample t-test

data:  bar.df.wash.j$in.ph and bar.df.nowash.j$in.ph
t = 0.4192, df = 14.86, p-value = 0.6811
alternative hypothesis: true difference in means is not equal to 0
95 percent confidence interval:
 -0.04196  0.06248
sample estimates:
mean of x mean of y
   0.2208    0.2105
```

Very little to report there. That is a question that should be asked a bit more elegantly. In our case, we collected the data just in case it showed up as important.

One last barplot to show whether or not our phones are more indicative of our own microbiome. The workflow will be pretty much the same, but we are picking out:

- the similarities of each person's index finger compared to their own phone, and
- each person's finger compared to the average distance to everyone else's phone

```
dismat <- as.matrix(dis)
dismat <- 1-dismat

bar.df2 <- data.frame(matrix(0,17,2))
names(bar.df2) <- c('same.in.ph', 'others.in.ph')
row.names(bar.df2) <- unique(map$individ)

for(i in 1:nrow(bar.df2)) {
    bar.df2[i, 1] <- dismat[which(map$individ == row.names(bar.df2)[i] &
                                    map$location == 'index'),
                              which(map$individ == row.names(bar.df2)[i] &
                                    map$location == 'phone')]
    bar.df2[i, 2] <- mean(dismat[which(map$individ == row.names(bar.df2)[i] &
                                    map$location == 'index'),
                              which(map$individ != row.names(bar.df2)[i] &
                                    map$location == 'phone')])
  }

bar.summary2 <- data.frame(
                cbind(apply(bar.df2, 2, mean),
                     (apply(bar.df2, 2, sd)/sqrt(nrow(bar.df2)))))

bar.summary2[, 3] <- bar.summary2[, 1]-bar.summary2[, 2]
bar.summary2[, 4] <- bar.summary2[, 1]+bar.summary2[, 2]
names(bar.summary2) <- c('mean', 'se', 'se.lo', 'se.hi')
bar.others <- bar.summary2
bar.others.df <- bar.df2
```

And make the plot.

```
# pdf('resemblePhone.pdf', width=2.5, height=3)
# postscript('resemblePhone.eps', width=2.5, height=3)

par(mar=c(3, 4.5, 2, 1), las=1, col.axis='gray20', col.lab='gray20', fg='gray20')
mids <- barplot(bar.others$mean, las=1,
    border='transparent', axes=FALSE, ylim=c(0,.3), yaxs='i',
    # ylab='Percent of species in common')
  ylab='')
mtext('Jaccard Similarity\n(as % of shared OTUs)', side=2, line=2.2, las=0)
abline(h=seq(0, .3, .05), col='white', lwd=1)
mtext(c('index &\nown\nphone', 'index &\nother\nphones'),
    side=1, line=1.6, at=c(mids), cex=.8, col='gray20')
axis(2, col='gray20', col.ticks='gray20',
    at=c(0,.1,.2,.3), labels=c(0,10,20,'30%'))
arrows(mids, bar.others$se.lo, mids, bar.others$se.hi, code=3,
    angle=90, length=.05, col='gray40')
mtext('Does your phone\nresemble you?', font=2, col='gray20')
```

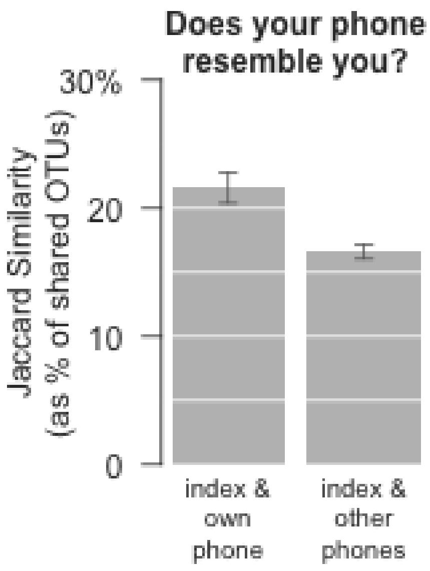


Figure 4: plot of chunk makePersonToPhoneBarplot

```
# dev.off()
```

And is that significant?

```
apply(bar.others.df, 2, mean)
```

```
  same.in.ph others.in.ph
      0.2159       0.1661
```

```
t.test(bar.others.df$same.in.ph, bar.others.df$others.in.ph, paired=TRUE)
```

```

    Paired t-test

data:  bar.others.df$same.in.ph and bar.others.df$others.in.ph
t = 4.286, df = 16, p-value = 0.0005674
alternative hypothesis: true difference in means is not equal to 0
95 percent confidence interval:
 0.02519 0.07451
sample estimates:
mean of the differences
                0.04985
```

So yes, your phone might be able to identify you. Or in other words, we see some evidence that the microbial assemblages on our phones are perhaps extensions of our own, and that they are to some degree personalized to us!

But this distance might just be driven by women, so quick check to see if this result varies by gender. It is lower power, so can't really be compared directly to the test above, but gives us an idea.

```r
bar.others.f.df <- bar.others.df[which(map[index, 'gender'] == 'f'), ]

bar.summary.f <- data.frame(
            cbind(apply(bar.others.f.df, 2, mean),
                     (apply(bar.others.f.df, 2, sd)/sqrt(nrow(bar.others.f.df)))))

bar.summary.f[, 3] <- bar.summary.f[, 1]-bar.summary.f[, 2]
bar.summary.f[, 4] <- bar.summary.f[, 1]+bar.summary.f[, 2]
names(bar.summary.f) <- c('mean', 'se', 'se.lo', 'se.hi')
```

```r
bar.others.m.df <- bar.others.df[which(map[index, 'gender'] == 'm'), ]

bar.summary.m <- data.frame(
            cbind(apply(bar.others.m.df, 2, mean),
                     (apply(bar.others.m.df, 2, sd)/sqrt(nrow(bar.others.m.df)))))

bar.summary.m[, 3] <- bar.summary.m[, 1]-bar.summary.m[, 2]
bar.summary.m[, 4] <- bar.summary.m[, 1]+bar.summary.m[, 2]
names(bar.summary.m) <- c('mean', 'se', 'se.lo', 'se.hi')
```

And make the plots.

```r
# pdf('resembleWomen.pdf', width=2.5, height=3)
par(mar=c(3, 4.5, 2, 1), las=1, col.axis='gray20', col.lab='gray20', fg='gray20')
mids <- barplot(bar.summary.f$mean, las=1,
    border='transparent', axes=FALSE, ylim=c(0,.3), yaxs='i',
  ylab='')
mtext('Jaccard Similarity\n(as % of shared OTUs)', side=2, line=2.2, las=0)
abline(h=seq(0, .3, .05), col='white', lwd=1)
mtext(c('index &\nown\nphone', 'index &\nother\nphones'),
    side=1, line=1.6, at=c(mids), cex=.8, col='gray20')
axis(2, col='gray20', col.ticks='gray20',
    at=c(0,.1,.2,.3), labels=c(0,10,20,'30%'))
arrows(mids, bar.summary.f$se.lo, mids, bar.summary.f$se.hi, code=3,
    angle=90, length=.05, col='gray40')
mtext("Do women resemble\ntheir phones?", font=2, col='gray20')
```

```r
# dev.off()

# pdf('resembleMen.pdf', width=2.5, height=3)
par(mar=c(3, 4.5, 2, 1), las=1, col.axis='gray20', col.lab='gray20', fg='gray20')
mids <- barplot(bar.summary.m$mean, las=1,
  border='transparent', axes=FALSE, ylim=c(0,.3), yaxs='i',
  ylab='')
mtext('Jaccard Similarity\n(as % of shared OTUs)', side=2, line=2.2, las=0)
abline(h=seq(0, .3, .05), col='white', lwd=1)
mtext(c('index &\nown\nphone', 'index &\nother\nphones'),
    side=1, line=1.6, at=c(mids), cex=.8, col='gray20')
axis(2, col='gray20', col.ticks='gray20',
    at=c(0,.1,.2,.3), labels=c(0,10,20,'30%'))
arrows(mids, bar.summary.m$se.lo, mids, bar.summary.m$se.hi, code=3,
```

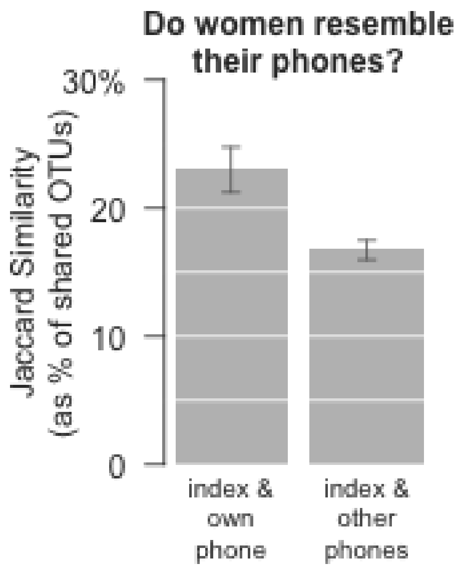


Figure 5: plot of chunk makeGenderToPhoneBarplot

```
    angle=90, length=.05, col='gray40')
mtext("Do men resemble\ntheir phones?", font=2, col='gray20')
```

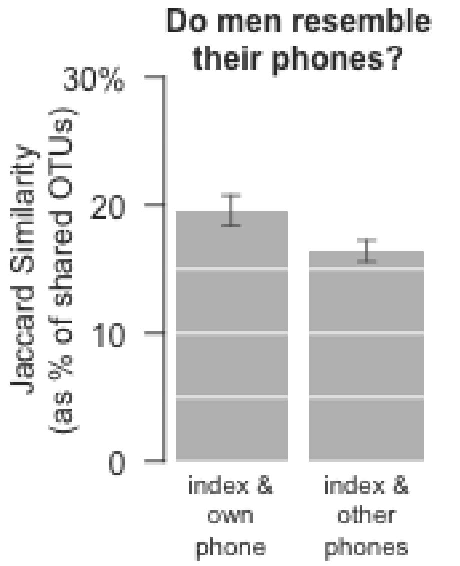


Figure 6: plot of chunk makeGenderToPhoneBarplot

```
# dev.off()
```

And is that significant for both groups?

```r
diff.f <- round(diff(c(apply(bar.others.f.df, 2, mean))) * (-100), digits=2)
t.test(bar.others.f.df$same.in.ph, bar.others.f.df$others.in.ph, paired=TRUE)
```

```
    Paired t-test

data:  bar.others.f.df$same.in.ph and bar.others.f.df$others.in.ph
t = 3.691, df = 9, p-value = 0.004987
alternative hypothesis: true difference in means is not equal to 0
95 percent confidence interval:
 0.02426 0.10106
sample estimates:
mean of the differences
                0.06266
```

```r
diff.m <- round(diff(c(apply(bar.others.m.df, 2, mean))) * (-100), digits=2)
t.test(bar.others.m.df$same.in.ph, bar.others.m.df$others.in.ph, paired=TRUE)
```

```
    Paired t-test

data:  bar.others.m.df$same.in.ph and bar.others.m.df$others.in.ph
t = 2.464, df = 6, p-value = 0.04882
alternative hypothesis: true difference in means is not equal to 0
95 percent confidence interval:
 0.0002245 0.0628777
sample estimates:
mean of the differences
                0.03155
```

Yes - both sexes share, on average, more with their own phones than with everyone else's. Men share about 3.16% percent more taxa with their own than with other phones, while women share 6.27% more than they share with other people's phones.

Here's the figure that goes in the manuscript.

```r
# pdf('resemblePhone.pdf', width=5, height=4)
# postscript('resemblePhone.eps', width=5, height=4)

line=2.6
xl1 <- 0.2
xl2 <- 2.4

layout(matrix(c(1:3), 1, 3), widths=c(1.35, 1, 1))
par(mar=c(4, 5, 2, 1), las=1, col.axis='gray20', col.lab='gray20', fg='gray20')
mids <- barplot(bar.others$mean, las=1,
  border='transparent', axes=FALSE, ylim=c(0,.3), yaxs='i',
    ylab='', col=c('gray80', 'gray50'))
mtext('Jaccard Similarity\n(as % of shared OTUs)', side=2, line=2.2, las=0, at=.14)
abline(h=seq(0, .3, .05), col='white', lwd=1)
mtext(c('index &\nown\nphone', 'index &\nother\nphones'),
    side=1, line=line, at=c(mids), cex=.8, col='gray20')
```

```
axis(2, col='gray20', col.ticks='gray20',
    at=c(0,.1,.2,.25), labels=c(0,10,20,'25%'))
arrows(mids, bar.others$se.lo, mids, bar.others$se.hi, code=3,
    angle=90, length=.05, col='gray40')
mtext('All\nparticipants', font=2, col='gray20', line=-2)
par(xpd=TRUE)
segments(xl1, 0, xl2, 0, col='gray30')

par(mar=c(4, 1, 2, 1), las=1, col.axis='gray20',
    col.lab='gray20', fg='gray20', xpd=FALSE)
mids <- barplot(bar.summary.f$mean, las=1,
  border='transparent', axes=FALSE, ylim=c(0,.3), yaxs='i',
  ylab='', col=c('gray80', 'gray50'))
abline(h=seq(0, .3, .05), col='white', lwd=1)
mtext(c('index &\nown\nphone', 'index &\nother\nphones'),
    side=1, line=line, at=c(mids), cex=.8, col='gray20')
arrows(mids, bar.summary.f$se.lo, mids, bar.summary.f$se.hi, code=3,
    angle=90, length=.05, col='gray40')
mtext("Only\nwomen", font=2, col='gray20', line=-2)
par(xpd=TRUE)
segments(xl1, 0, xl2, 0, col='gray30')

par(mar=c(4, 1, 2, 1), las=1, col.axis='gray20', col.lab='gray20', fg='gray20')
mids <- barplot(bar.summary.m$mean, las=1,
  border='transparent', axes=FALSE, ylim=c(0,.3), yaxs='i',
  ylab='', col=c('gray80', 'gray50'))
abline(h=seq(0, .3, .05), col='white', lwd=1)
mtext(c('index &\nown\nphone', 'index &\nother\nphones'),
    side=1, line=line, at=c(mids), cex=.8, col='gray20')
arrows(mids, bar.summary.m$se.lo, mids, bar.summary.m$se.hi, code=3,
    angle=90, length=.05, col='gray40')
mtext("Only\nmen", font=2, col='gray20', line=-2)
par(xpd=TRUE)
segments(xl1, 0, xl2, 0, col='gray30')
```

```
# dev.off()
```

That includes the very rare long tail of OTUs in the dataset. How about the most common OTUs?

```
sortSums <- sort(colSums(rw.7k))/sum(rw.7k)
plot(rev(sortSums), las=1, pch='.', cex=3, bty='l')
```

```
the.1percent <- length(which(sortSums > .001))
```

It looks like 79 of the OTUs represent more than 0.1% of the dataset. So the next move is to see how many of these (for each person) are shared between index and phone, and also between index and thumb.

```
people <- unique(map$individ)
lpeople <- length(people)
perShared <- data.frame(sharedPhones=rep(0, lpeople),
```

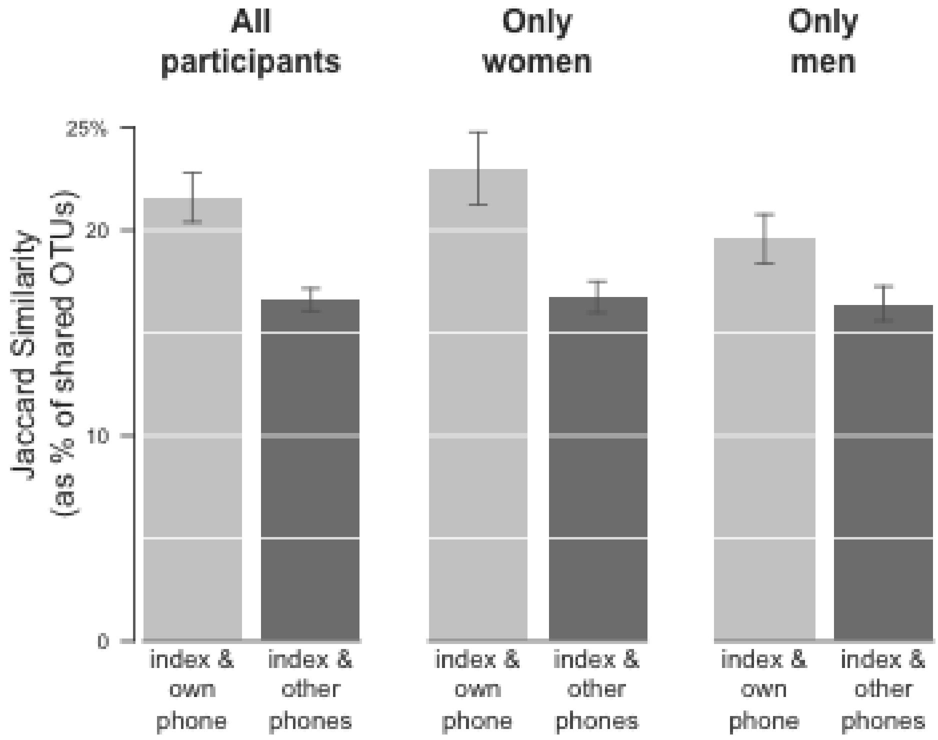


Figure 7: plot of chunk makeLongPersonToPhoneBarplot

```
                        sharedFingers=rep(0, lpeople),
                        totalPhones=rep(0, lpeople),
                        totalFingers=rep(0, lpeople),
                        row.names=people)
for (i in 1:length(people)) {
  # phones and fingers
  total.1percent <- rw.7k[map$individ == people[i] &
                          map$location %in% c('index', 'phone'), ]
  just.1percent <- total.1percent[, which((colSums(total.1percent)/
                                          sum(total.1percent)) > 0.001)]
  perShared$totalPhones[i] <- dim(just.1percent)[2]
  perShared$sharedPhones[i] <- length(which(colSums(just.1percent>0) == 2))/
                           perShared$totalPhones[i]
  # just fingers
  total.1fingers <- rw.7k[map$individ == people[i] &
                          map$location %in% c('index', 'thumb'), ]
  just.1fingers <- total.1fingers[, which((colSums(total.1fingers)/
                                          sum(total.1fingers)) > 0.001)]
  perShared$totalFingers[i] <- dim(just.1fingers)[2]
  perShared$sharedFingers[i] <- length(which(colSums(just.1fingers>0) == 2))/
                           perShared$totalFingers[i]
  }
# perShared

pxtable(data.frame(rbind(apply(perShared, 2, mean),
                 apply(perShared, 2, se)),
```

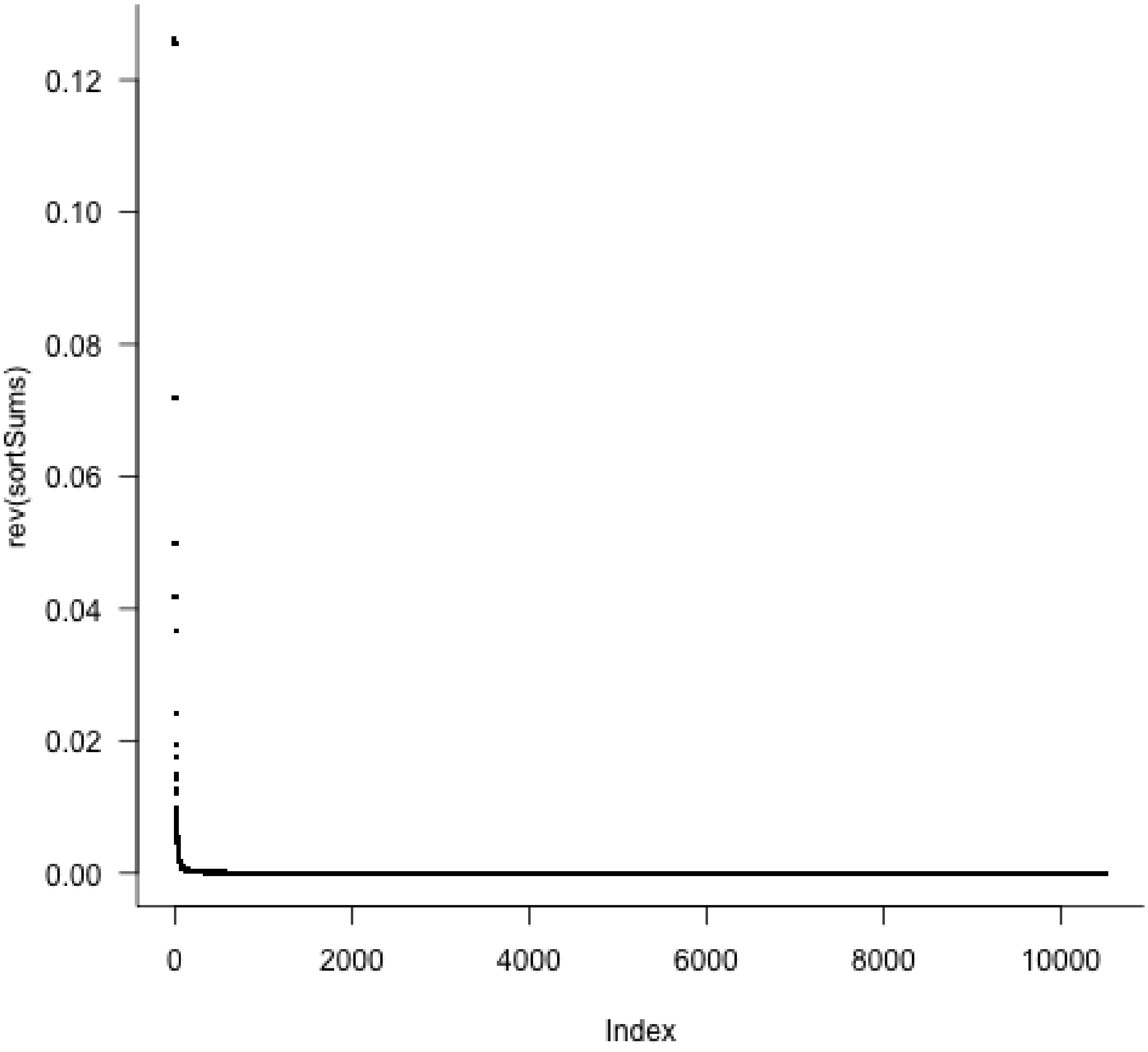


Figure 8: plot of chunk thePoint1percent

```
         row.names=c('mean', 'se')),
      capt='Shared OTUs (more than 0.1 percent relative abundance)')
```

| | sharedPhones | sharedFingers | totalPhones | totalFingers |
|---|---|---|---|---|
| mean | 0.82 | 0.96 | 70.29 | 60.94 |
| se | 0.03 | 0.02 | 3.80 | 2.82 |

Table 6: Shared OTUs (more than 0.1 percent relative abundance)

---

### Community differences - Ordination and Discriminant Analysis

Do the fingers of men and women harbor different types of bacteria? Previous research says yes. In the current study, some people washed hands and some didn't. So we should find out if we have a balanced study (i.e., relatively even numbers in all four categories?). The answer is, of course, somewhat funny, though not *significantly* funny.

```
gender.wash <- table(map$gender, map$wash)
cst <- chisq.test(gender.wash)
cst
```

```

    Pearson's Chi-squared test with Yates' continuity correction

data:  gender.wash
X-squared = 0.8503, df = 1, p-value = 0.3565
```

```
cst$observed
```

```

     y  n
  f 18 12
  m  9 12
```

```
cst$expected
```

```

        y      n
  f 15.88 14.118
  m 11.12  9.882
```

```
rm(cst, gender.wash)
```

Anyway, the sample is reasonably well balanced. PERMANOVA tests like `adonis` are not necessarily robust to big inbalances, but probably not a problem for us.

We'll use the Canberra distance, since we expect most of the abundant taxa to overlap - we are interested in differences among the relatively rare OTUs. Compared to the easily interpretable Jaccard index used above,

these sophisticated dissimilarities tend to be more mathematically satisfying for ordination. If we look at an NMDS of all samples, it looks like there is a separation between fingers and phones, but not between finger types. Not too surprising given what we saw in the bar plots above. Notice that the ordination gets put into a generic object `n` to save on typing but also to make it easier to switch distance matrices or nmds objects later.

```
rw.7k.can <- vegdist(rw.7k, 'canberra')
```

Create NMDS.

```
rw.7k.nmds.can <- bestnmds(rw.7k.can, k=2)
```

If we emphasize gender, it seems that men and women fall in different parts of the plot, and their communities, when considering all samples, are very significantly different.

```
rw.7k.nmds.can$stress
```

```
[1] 20.69
```

```
n <- rw.7k.nmds.can

par(mfrow=c(1,1))
par(mar=c(2,2,1,1), las=0)
plot(n$points, pch=16, cex=3, ann=FALSE, xaxt='n', yaxt='n',
  col=ifelse(map$location == 'phone', rgb(0,0,0, .5),
    ifelse(map$location == 'index',
            rgb(100/255, 149/255, 237/255, .8), # cornflowerblue
            rgb(255/255, 140/255, 0/255, .8)))) # darkorange
mtext('NMDS 1', side=1, line=.0, col='gray40', adj=1)
mtext('NMDS 2', side=2, line=.0, col='gray40', adj=1)
legend('topright', legend=c('index', 'thumb', 'phone'),
    pch=16, col=c('cornflowerblue', 'darkorange', 'gray40'),
    bty='n', pt.cex=1.5)
```

Earlier, we created sets of variables that combine sample location with gender - this now allows the use of ellipses to visualize confidence intervals in the ordination.

It would be ideal if we could get most information only from sampling a single finger in future cell phone monitoring studies, instead of looking at both index fingers and thumbs. This ordination displays both fingers and phones.

```
# pdf('ordinationGenderBothFingers.pdf', height=4, width=8)
par(mfrow=c(1,2))
par(mar=c(2,2,2,1), las=0)
plot(n$points, type='n', ann=FALSE, xaxt='n', yaxt='n')
mtext('NMDS 1', side=1, line=.3, col='gray40', adj=1)
mtext('NMDS 2', side=2, line=.0, col='gray40', adj=1)
points(n$points[intersect(m,finger), ], pch=21, cex=2, col='gray', bg='white')
points(n$points[intersect(m,p), ], pch=21, cex=2, col='gray', bg='white')
ordiellipse(n, groups=map$loc.gen, show.groups='phone.f',
            draw='polygon', col='gray80')
ordiellipse(n, groups=map$loc.gen, show.groups='phone.f',
```

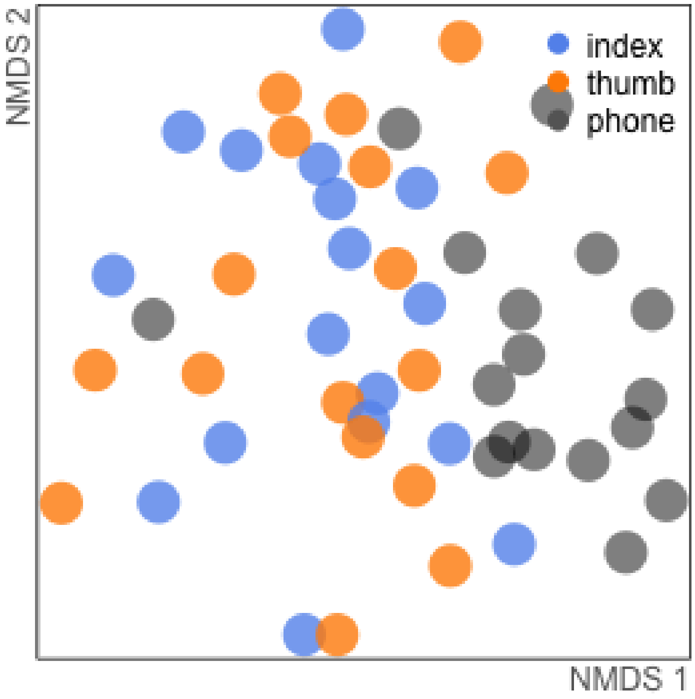


Figure 9: plot of chunk plotNMDS

```
            draw='lines', lwd=1.2, col='gray80')
ordiellipse(n, groups=map$loc.gen2, show.groups='finger.f',
            draw='polygon', col='cornflowerblue')
ordiellipse(n, groups=map$loc.gen2, show.groups='finger.f',
            draw='lines', lwd=1.2, col='gray80')
points(n$points[intersect(f,finger), ], pch=21, cex=2.5, col='gray30',
      bg=rgb(100/255, 149/255, 237/255, .8)) # cornflowerblue
points(n$points[intersect(f,p), ], pch=21, cex=2.5, col='gray30',
      bg=rgb(0,0,0, .5))
mtext('(a) Females', line=.2, font=2, cex=1.5, adj=0)

par(mar=c(2,1,2,2), las=0)
plot(n$points, type='n', ann=FALSE, xaxt='n', yaxt='n')
mtext('NMDS 1', side=1, line=.3, col='gray40', adj=1)
#mtext('NMDS 2', side=2, line=.0, col='gray40', adj=1)
points(n$points[intersect(f,finger), ], pch=21, cex=2, col='gray', bg='white')
points(n$points[intersect(f,p), ], pch=21, cex=2, col='gray', bg='white')
ordiellipse(n, groups=map$loc.gen, show.groups='phone.m',
            draw='polygon', col='gray80')
ordiellipse(n, groups=map$loc.gen, show.groups='phone.m',
            draw='lines', lwd=1.2, col='gray80')
ordiellipse(n, groups=map$loc.gen2, show.groups='finger.m',
            draw='polygon', col='cornflowerblue')
ordiellipse(n, groups=map$loc.gen2, show.groups='finger.m',
            draw='lines', lwd=1.2, col='gray80')
points(n$points[intersect(m,finger), ], pch=21, cex=2.5, col='gray30',
```

```
      bg=rgb(100/255, 149/255, 237/255, .8)) # cornflowerblue
points(n$points[intersect(m,p), ], pch=21, cex=2.5, col='gray30',
      bg=rgb(0,0,0, .5))
# text(5,5,'Males', font=2, cex=2, col='gray40')
legend('bottomright', legend=c('phone', 'finger'), pch=21, bty='n', y.intersp=.9,
       pt.bg=c('gray40', 'cornflowerblue'), col='gray30', cex=1, pt.cex=1.5)
mtext('(b) Males', line=.2, font=2, cex=1.5, adj=0)
```

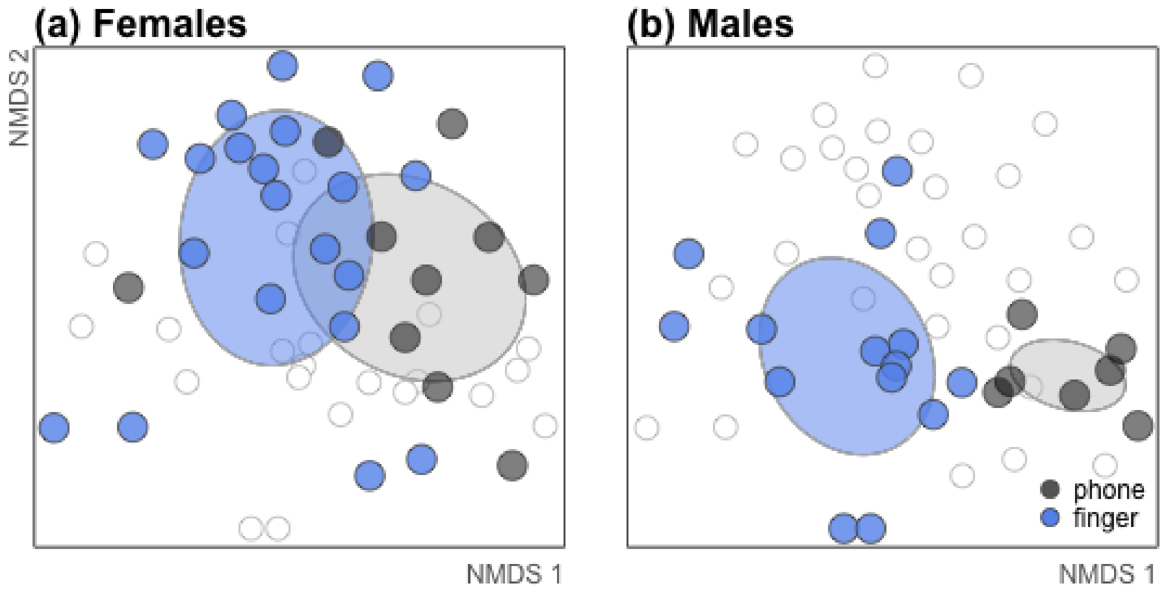


Figure 10: plot of chunk genderFingerNMDS

```
# dev.off()
```

This version uses only index fingers compared to phones.

```
# pdf('ordinationGenderIndex.pdf', height=4, width=8)
# postscript('ordinationGenderIndex.eps', height=4, width=8)

par(mfrow=c(1,2))
par(mar=c(2,2,2,1), las=0)
plot(n$points, type='n', ann=FALSE, xaxt='n', yaxt='n')
mtext('NMDS 1', side=1, line=.3, col='gray40', adj=1)
mtext('NMDS 2', side=2, line=.0, col='gray40', adj=1)
points(n$points[intersect(m,index), ], pch=21, cex=2, col='gray', bg='white')
points(n$points[intersect(m,p), ], pch=21, cex=2, col='gray', bg='white')
ordiellipse(n, groups=map$loc.gen, show.groups='phone.f',
            draw='polygon', col='gray80')
ordiellipse(n, groups=map$loc.gen, show.groups='phone.f',
            draw='lines', lwd=1.2, col='gray80')
ordiellipse(n, groups=map$loc.gen, show.groups='index.f',
            draw='polygon', col='cornflowerblue')
ordiellipse(n, groups=map$loc.gen, show.groups='index.f',
```

```
            draw='lines', lwd=1.2, col='gray80')
points(n$points[intersect(f,index), ], pch=21, cex=2.5, col='gray30',
      bg=rgb(100/255, 149/255, 237/255, .8)) # cornflowerblue
points(n$points[intersect(f,p), ], pch=21, cex=2.5, col='gray30',
      bg=rgb(0,0,0, .5))
mtext('(a) Females', line=.2, font=2, cex=1.5, adj=0)

par(mar=c(2,1,2,2), las=0)
plot(n$points, type='n', ann=FALSE, xaxt='n', yaxt='n')
mtext('NMDS 1', side=1, line=.3, col='gray40', adj=1)
points(n$points[intersect(f,index), ], pch=21, cex=2, col='gray', bg='white')
points(n$points[intersect(f,p), ], pch=21, cex=2, col='gray', bg='white')
ordiellipse(n, groups=map$loc.gen, show.groups='phone.m',
            draw='polygon', col='gray80')
ordiellipse(n, groups=map$loc.gen, show.groups='phone.m',
            draw='lines', lwd=1.2, col='gray80')
ordiellipse(n, groups=map$loc.gen, show.groups='index.m',
            draw='polygon', col='cornflowerblue')
ordiellipse(n, groups=map$loc.gen, show.groups='index.m',
            draw='lines', lwd=1.2, col='gray80')
points(n$points[intersect(m,index), ], pch=21, cex=2.5, col='gray30',
      bg=rgb(100/255, 149/255, 237/255, .8)) # cornflowerblue
points(n$points[intersect(m,p), ], pch=21, cex=2.5, col='gray30',
      bg=rgb(0,0,0, .5))
# text(5,5,'Males', font=2, cex=2, col='gray40')
legend('bottomright', legend=c('phone', 'index'), pch=21, bty='n', y.intersp=.9,
       pt.bg=c('gray40', 'cornflowerblue'), col='gray30', cex=1, pt.cex=1.5)
mtext('(b) Males', line=.2, font=2, cex=1.5, adj=0)
```

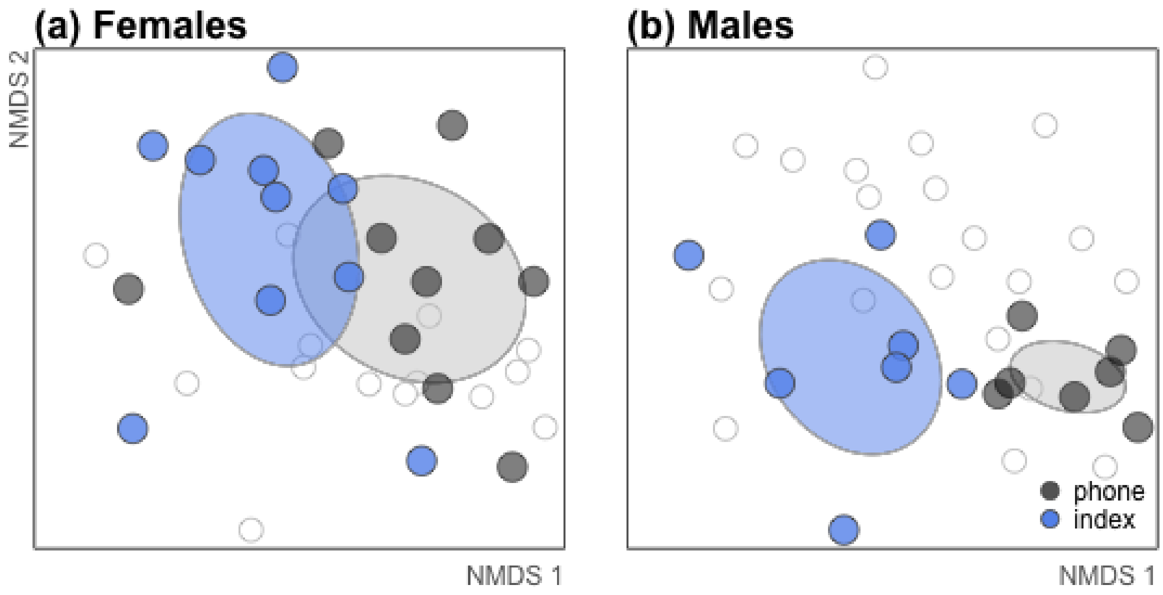


Figure 11: plot of chunk genderIndexNMDS

```
# dev.off()
```

It seems clear that women and men are falling out in different parts of the ordination. And in fact the difference is significant for both phones and fingers - gender makes a difference.

```
can.phones <- as.dist(as.matrix(rw.7k.can)[p, p])
can.fingers <- as.dist(as.matrix(rw.7k.can)[finger, finger])
can.index <- as.dist(as.matrix(rw.7k.can)[index, thumb])
can.thumb <- as.dist(as.matrix(rw.7k.can)[thumb, thumb])

map.phones <- map[p, ]
map.fingers <- map[finger, ]
map.index <- map[index, ]
map.thumb <- map[thumb, ]

adonisAllGender <- adonis(rw.7k.can ~ map$gender)$aov.tab
adonisPhoneGender <- adonis(can.phones ~ map.phones$gender)$aov.tab
adonisFingerGender <- adonis(can.fingers ~ map.fingers$gender)$aov.tab
adonisIndexGender <- adonis(can.index ~ map.index$gender)$aov.tab
adonisThumbGender <- adonis(can.thumb ~ map.thumb$gender)$aov.tab

#print(xtable(adonisAllGender))#, type='html')
pxtable(adonisAllGender, capt='Gender difference for all samples together?')
```

| | Df | SumsOfSqs | MeanSqs | F.Model | R2 | Pr(>F) |
|---|---|---|---|---|---|---|
| map$gender | 1 | 0.61 | 0.61 | 1.54 | 0.03 | 0.0010 |
| Residuals | 49 | 19.59 | 0.40 | | 0.97 | |
| Total | 50 | 20.20 | | | 1.00 | |

Table 7: Gender difference for all samples together?

```
pxtable(adonisFingerGender, capt='Gender difference for both fingers together?')
```

| | Df | SumsOfSqs | MeanSqs | F.Model | R2 | Pr(>F) |
|---|---|---|---|---|---|---|
| map.fingers$gender | 1 | 0.58 | 0.58 | 1.45 | 0.04 | 0.0020 |
| Residuals | 32 | 12.70 | 0.40 | | 0.96 | |
| Total | 33 | 13.28 | | | 1.00 | |

Table 8: Gender difference for both fingers together?

```
pxtable(adonisPhoneGender, capt='Gender difference for just phones?')
```

| | Df | SumsOfSqs | MeanSqs | F.Model | R2 | Pr(>F) |
|---|---|---|---|---|---|---|
| map.phones$gender | 1 | 0.47 | 0.47 | 1.20 | 0.07 | 0.0070 |
| Residuals | 15 | 5.90 | 0.39 | | 0.93 | |
| Total | 16 | 6.37 | | | 1.00 | |

Table 9: Gender difference for just phones?

```r
pxtable(adonisIndexGender, capt='Gender difference for just index fingers?')
```

| | Df | SumsOfSqs | MeanSqs | F.Model | R2 | Pr(>F) |
|---|---|---|---|---|---|---|
| map.index$gender | 1 | 0.45 | 0.45 | 1.13 | 0.07 | 0.0330 |
| Residuals | 15 | 6.01 | 0.40 | | 0.93 | |
| Total | 16 | 6.46 | | | 1.00 | |

Table 10: Gender difference for just index fingers?

```r
pxtable(adonisThumbGender, capt='Gender difference for just thumbs?')
```

| | Df | SumsOfSqs | MeanSqs | F.Model | R2 | Pr(>F) |
|---|---|---|---|---|---|---|
| map.thumb$gender | 1 | 0.46 | 0.46 | 1.14 | 0.07 | 0.0270 |
| Residuals | 15 | 6.03 | 0.40 | | 0.93 | |
| Total | 16 | 6.48 | | | 1.00 | |

Table 11: Gender difference for just thumbs?

Phones, maybe - but also underpowered. Fingers, definitely, but this includes all fingers together which is pseudoreplication. Best to rely on just one finger.

Do index fingers tell the whole story? Or are both fingers together more powerful? It is a tough question because it is an unbalanced comparison, but the nmds above makes it clear that the significant separation from phones is evident even when only using an index and not grouping both fingers.

```
can.index.phone.f <- as.dist(as.matrix(rw.7k.can)[intersect(c(index,p),f),
                                                  intersect(c(index,p),f)])
map.index.phone.f <- map[intersect(c(index,p),f), ]
can.index.phone.m <- as.dist(as.matrix(rw.7k.can)[intersect(c(index,p),m),
                                                  intersect(c(index,p),m)])
map.index.phone.m <- map[intersect(c(index,p),m), ]

can.thumb.phone.f <- as.dist(as.matrix(rw.7k.can)[intersect(c(thumb,p),f),
                                                  intersect(c(thumb,p),f)])
map.thumb.phone.f <- map[intersect(c(index,p),f), ]
can.thumb.phone.m <- as.dist(as.matrix(rw.7k.can)[intersect(c(thumb,p),m),
                                                  intersect(c(thumb,p),m)])
map.thumb.phone.m <- map[intersect(c(thumb,p),m), ]

can.finger.phone.f <- as.dist(as.matrix(rw.7k.can)[f, f])
map.finger.phone.f <- map[f, ]
can.finger.phone.m <- as.dist(as.matrix(rw.7k.can)[m, m])
map.finger.phone.m <- map[m, ]


adonisIndexPhoneF <- adonis(can.index.phone.f ~ map.index.phone.f$location)$aov.tab
adonisIndexPhoneM <- adonis(can.index.phone.m ~ map.index.phone.m$location)$aov.tab
adonisThumbPhoneF <- adonis(can.thumb.phone.f ~ map.thumb.phone.f$location)$aov.tab
adonisThumbPhoneM <- adonis(can.thumb.phone.m ~ map.thumb.phone.m$location)$aov.tab
adonisFingerPhoneF <- adonis(can.finger.phone.f ~ map.finger.phone.f$location2)$aov.tab
adonisFingerPhoneM <- adonis(can.finger.phone.m ~ map.finger.phone.m$location2)$aov.tab

pxtable(adonisIndexPhoneF, capt="Women's phones different from index fingers?")
```

| | Df | SumsOfSqs | MeanSqs | F.Model | R2 | Pr(>F) |
|---|---|---|---|---|---|---|
| map.index.phone.f$location | 1 | 0.41 | 0.41 | 1.01 | 0.05 | 0.3270 |
| Residuals | 18 | 7.28 | 0.40 | | 0.95 | |
| Total | 19 | 7.69 | | | 1.00 | |

Table 12: Women's phones different from index fingers?

```
pxtable(adonisIndexPhoneM, capt="Men's phones different from index fingers?")
```

| | Df | SumsOfSqs | MeanSqs | F.Model | R2 | Pr(>F) |
|---|---|---|---|---|---|---|
| map.index.phone.m$location | 1 | 0.50 | 0.50 | 1.30 | 0.10 | 0.0010 |
| Residuals | 12 | 4.61 | 0.38 | | 0.90 | |
| Total | 13 | 5.11 | | | 1.00 | |

Table 13: Men's phones different from index fingers?

```
pxtable(adonisThumbPhoneF, capt="Women's phones different from thumbs?")
```

| | Df | SumsOfSqs | MeanSqs | F.Model | R2 | Pr(>F) |
|---|---|---|---|---|---|---|
| map.thumb.phone.f$location | 1 | 0.41 | 0.41 | 1.00 | 0.05 | 0.4550 |
| Residuals | 18 | 7.29 | 0.40 | | 0.95 | |
| Total | 19 | 7.69 | | | 1.00 | |

Table 14: Women's phones different from thumbs?

```
pxtable(adonisThumbPhoneM, capt="Men's phones different from thumbs?")
```

| | Df | SumsOfSqs | MeanSqs | F.Model | R2 | Pr(>F) |
|---|---|---|---|---|---|---|
| map.thumb.phone.m$location | 1 | 0.50 | 0.50 | 1.30 | 0.10 | 0.0010 |
| Residuals | 12 | 4.64 | 0.39 | | 0.90 | |
| Total | 13 | 5.14 | | | 1.00 | |

Table 15: Men's phones different from thumbs?

```
pxtable(adonisFingerPhoneF, capt="Women's phones different from both fingers together?")
```

| | Df | SumsOfSqs | MeanSqs | F.Model | R2 | Pr(>F) |
|---|---|---|---|---|---|---|
| map.finger.phone.f$location2 | 1 | 0.43 | 0.43 | 1.06 | 0.04 | 0.1680 |
| Residuals | 28 | 11.28 | 0.40 | | 0.96 | |
| Total | 29 | 11.70 | | | 1.00 | |

Table 16: Women's phones different from both fingers together?

```
pxtable(adonisFingerPhoneM, capt="Men's phones different from both fingers together?")
```

| | Df | SumsOfSqs | MeanSqs | F.Model | R2 | Pr(>F) |
|---|---|---|---|---|---|---|
| map.finger.phone.m$location2 | 1 | 0.56 | 0.56 | 1.45 | 0.07 | 0.0010 |
| Residuals | 19 | 7.33 | 0.39 | | 0.93 | |
| Total | 20 | 7.89 | | | 1.00 | |

Table 17: Men's phones different from both fingers together?

Index fingers alone seem to explain the most variation. $R^2$ is higher, though the F values are slightly lower due to less statistical power. This indicates that index alone is a good finger to use in further studies. Additionally, women don't show a significant difference from the communities on their phones, while men do! Perhaps women will be easier to track by their phones? This contrasts slightly from the results above that say that both men and women share more OTUs with their phones than other people's phones. So this indicates that whether the phone can be used to track person microbiomes will depend on how we look at the data.

```r
ls()
```

```
 [1] "act.mean"               "act.se"
 [3] "act.table"              "act.table.10"
 [5] "act.taxo"               "act.taxo.10"
 [7] "adonisAllGender"        "adonisFingerGender"
 [9] "adonisFingerPhoneF"     "adonisFingerPhoneM"
[11] "adonisIndexGender"      "adonisIndexPhoneF"
[13] "adonisIndexPhoneM"      "adonisPhoneGender"
[15] "adonisThumbGender"      "adonisThumbPhoneF"
[17] "adonisThumbPhoneM"      "bar.df"
[19] "bar.df.female.j"        "bar.df.male.j"
[21] "bar.df.nowash.j"        "bar.df.wash.j"
[23] "bar.df2"                "bar.female.j"
[25] "bar.jac"                "bar.jac.df"
[27] "bar.male.j"             "bar.mf"
[29] "bar.nowash.j"           "bar.others"
[31] "bar.others.df"          "bar.others.f.df"
[33] "bar.others.m.df"        "bar.summary.f"
[35] "bar.summary.m"          "bar.summary2"
[37] "bar.wash.j"             "bar.wnw"
[39] "barname"                "can.finger.phone.f"
[41] "can.finger.phone.m"     "can.fingers"
[43] "can.index"              "can.index.phone.f"
[45] "can.index.phone.m"      "can.phones"
[47] "can.thumb"              "can.thumb.phone.f"
[49] "can.thumb.phone.m"      "cont"
[51] "cont.otus"              "cont.otus.names"
[53] "cont.table"             "cont3.otus"
[55] "diff.f"                 "diff.m"
[57] "dis"                    "dismat"
[59] "Evenness"               "f"
[61] "finger"                 "fir.mean"
[63] "fir.se"                 "fir.table"
[65] "fir.table.10"           "fir.taxo"
[67] "fir.taxo.10"            "font"
[69] "i"                      "index"
[71] "JacJFM"                 "just.1fingers"
[73] "just.1percent"          "line"
[75] "lpeople"                "m"
[77] "makeBarDF"              "makeBarSummary"
[79] "makeTaxo"               "map"
[81] "map.finger.phone.f"     "map.finger.phone.m"
[83] "map.fingers"            "map.index"
[85] "map.index.phone.f"      "map.index.phone.m"
[87] "map.phones"             "map.thumb"
[89] "map.thumb.phone.f"      "map.thumb.phone.m"
```

```
 [91] "medAbu5"                "medAbu5.f"
 [93] "medAbu5.fing"           "medAbu5.m"
 [95] "medAbu5.p"              "mids"
 [97] "n"                      "name.cex"
 [99] "p"                      "people"
[101] "perShared"              "ph"
[103] "ph.mean"                "ph.se"
[105] "pro.mean"               "pro.se"
[107] "pro.table"              "pro.table.10"
[109] "pro.taxo"               "pro.taxo.10"
[111] "pxtable"                "QiimeIn"
[113] "rw.7k"                  "rw.7k.can"
[115] "rw.7k.nmds.can"         "rw.7k.rel"
[117] "rw.big"                 "rw.table.noTop3control"
[119] "rw.taxo.7k"             "se"
[121] "sortSums"               "taxo"
[123] "test"                   "the.1percent"
[125] "thumb"                  "total.1fingers"
[127] "total.1percent"         "xl1"
[129] "xl2"                    "ylim"
```

```
save.image('phones_knitr.RData')
```

This cluttered workspace *should* have everything necessary to reproduce any analysis or figure separately without running the entire script.
