# Supplementary material for: Mobile phones carry the personal microbiome of their owners
Source: PeerJ. 2014 Jun 24;2:e447. doi: 10.7717/peerj.447 (PMC4081285; doi:10.7717/peerj.447)
Supplement: Data S2 — This document was added as a result of peer review. Herein, we explore the consequences of removing all potential laboratory contaminants as opposed to removing just the most obvious 3. This was produced using the knitr package in R. All data and corresponding scripts are available on GitHub: https://github.com/jfmeadow/Meadow_etal_Phones [file peerj-02-447-s002.pdf]

Phone Microbiome

Contamination analysis

James Meadow (jfmeadow at gmail dot com)

This document checks for the influence of potential laboratory contamination in the DNA sequence data used in https://github.com/jfmeadow/Meadow_etal_Phones. The initial treatment of the dataset is identical (and more thouroughly explained in the main script), but here the three different approaches to dealing with potential contamination are explored.

Getting data into shape

The first step is to set a random seed (so results are locked in with those reported in the manuscript) and load some necessary R packages and functions.

```
set.seed(42)
options(scipen=7)  # curtail scientific notation
library(vegan)
```

```
Loading required package: permute
Loading required package: lattice
This is vegan 2.0-10
```

```
library(labdsv)
```

```
Loading required package: mgcv
Loading required package: nlme
This is mgcv 1.7-28. For overview type 'help("mgcv-package")'.
Loading required package: MASS

Attaching package: 'labdsv'

The following object is masked from 'package:stats':

    density
```

```
library(miscTools)
library(xtable)
library(ggplot2)

# These options just for debugging - knitr is automatically in dir.
# setwd('~/Dropbox/rwjf/Meadow_etal_Phones/')
# load('phones.RData')

source('../functions.R')
```

The OTU table is brought in with a custom function `QiimeIn` that reads a classic OTU table and then splits it into a few useful pieces in a big list.

```
rw.list <- QiimeIn(file='../phones_otu_table.txt')
# removed comment character from first line so R takes it.
rw.map <- read.delim('../phones_map.txt', head=TRUE, row.names=1)
rw.big <- rw.list$Table
rw.taxo <- rw.list$Taxa
rm(rw.list)
```

Clean up OTU table.

```
row.names(rw.big) <- gsub('X', '', row.names(rw.big))
rw.big <- rw.big[row.names(rw.map), ]
```

Configure OTU table create three different versions.

To answer a question that arose during the first round of peer review, the contaminants are assessed for their influence over final results. So a table with contaminants, one minus only abundant contaminants, and one with all removed will be kept and assessed downstream.

First a few treatments for all 3 datasets:

- Remove plant sequences
- Do the same for mitochondrial sequences.
- Treat all three datasets similarily
- Remove OTUs that are represented by only 1 or 2 sequences - these lend little to community analysis and slow down the whole works.
- Rarefy to 2500 sequences per sample.

```
streptophyta <- grep('Streptophyta', rw.taxo$taxa.names)
mitochondria <- grep('mitochondria', rw.taxo$taxa.names)
rw.table.tmp <- rw.big[, -c(streptophyta, mitochondria)]
rw.table.tmp <- rw.table.tmp[, c(which(colSums(rw.table.tmp) > 2))]
rw.taxo.tmp <- rw.taxo[colnames(rw.table.tmp), ]
```

Pull out control samples to identify those OTUs found in controls.

```
cont <- grep('cont', row.names(rw.map))  # which samples are controls
cont.table <- rw.table.tmp[cont, ]  # otu table of only control rows
cont.otus <- which(colSums(cont.table) >0)  # which otus are present in controls
cont.otus.names <- colnames(cont.table)[cont.otus]  # what are their names
cont.taxo <- makeTaxo(taxo.in=rw.taxo.tmp$taxa.names, otu.table=cont.table)
```

```
Warning: no non-missing arguments to max; returning -Inf
```

```
cont3.otus <- which(colSums(cont.table)/sum(cont.table) > 0.05)  # pick out 3 big ones
```

Plot relative abundances of these potential contaminants to see how many are actually worrisome.

```r
plotY <- colSums(cont.table[, cont.otus]/sum(cont.table))
plotX <- colSums(rw.table.tmp[, cont.otus]/sum(rw.table.tmp))
plot(plotY ~ plotX,
     pch=21, bg=rgb(0,0,0,.3), cex=2, las=1,
     xlab='Rel Abundance in Experiment', ylab='Rel Abundance in Controls')
segments(0,0,1,1, lty=3, lwd=2, col='gray')
segments(0, .05, 1, .05, lty=1, lwd=2, col='tomato')
text(.12, .13, '1:1', font=3, col='gray30')
text(.1, .05, 'RA=0.05', font=3, pos=3, col='tomato')
text(plotX[names(cont3.otus)[1:2]], plotY[names(cont3.otus)[1:2]],
     cont.taxo[names(cont3.otus)[1:2], 'genus'], pos=c(3,1))
text(plotX[names(cont3.otus)[3]], plotY[names(cont3.otus)[3]],
     cont.taxo[names(cont3.otus)[3], 'family'], pos=3)
```

There is a clear cutoff at below 0.05 - these are the OTUs that are quite abundant in control samples that we should be worried about. Really, *Mycoplasma* seems like the biggest problem, but since the other two outliers are also very abundant in controls, it seems prudent to remove them anyway. The rest don't make much of a showing in controls but some are quite abundant in actual experimental samples. This might indicate some level of sample/barcode spillover which can happen lots of ways.

```r
rw.table.nocontrol <- rw.table.tmp[-cont, -cont.otus]  # take all contaminants out
rw.table.noTop3control <- rw.table.tmp[-cont, -cont3.otus]  # take out big 3
rw.table.allControl <- rw.table.tmp[-cont, ]  # leave in all contaminant OTUs
```

Rarefy all to 2500 seqs per sample.

```r
rw.25.NC <- rrarefy(rw.table.nocontrol, 2500)
rw.25.N3 <- rrarefy(rw.table.noTop3control, 2500)
rw.25.ALL <- rrarefy(rw.table.allControl, 2500)
```

Reconcile the taxonomic information in case it is required later.

```r
rw.taxo.NC <- rw.taxo.tmp[colnames(rw.25.NC), ]
rw.taxo.N3 <- rw.taxo.tmp[colnames(rw.25.N3), ]
rw.taxo.ALL <- rw.taxo.tmp[colnames(rw.25.ALL), ]
```

```r
jac.NC <- vegdist(rw.25.NC, 'jaccard')
jac.N3 <- vegdist(rw.25.N3, 'jaccard')
jac.ALL <- vegdist(rw.25.ALL, 'jaccard')
```

Jaccard distance

```r
can.NC <- vegdist(rw.25.NC, 'canberra')
can.N3 <- vegdist(rw.25.N3, 'canberra')
can.ALL <- vegdist(rw.25.ALL, 'canberra')
```

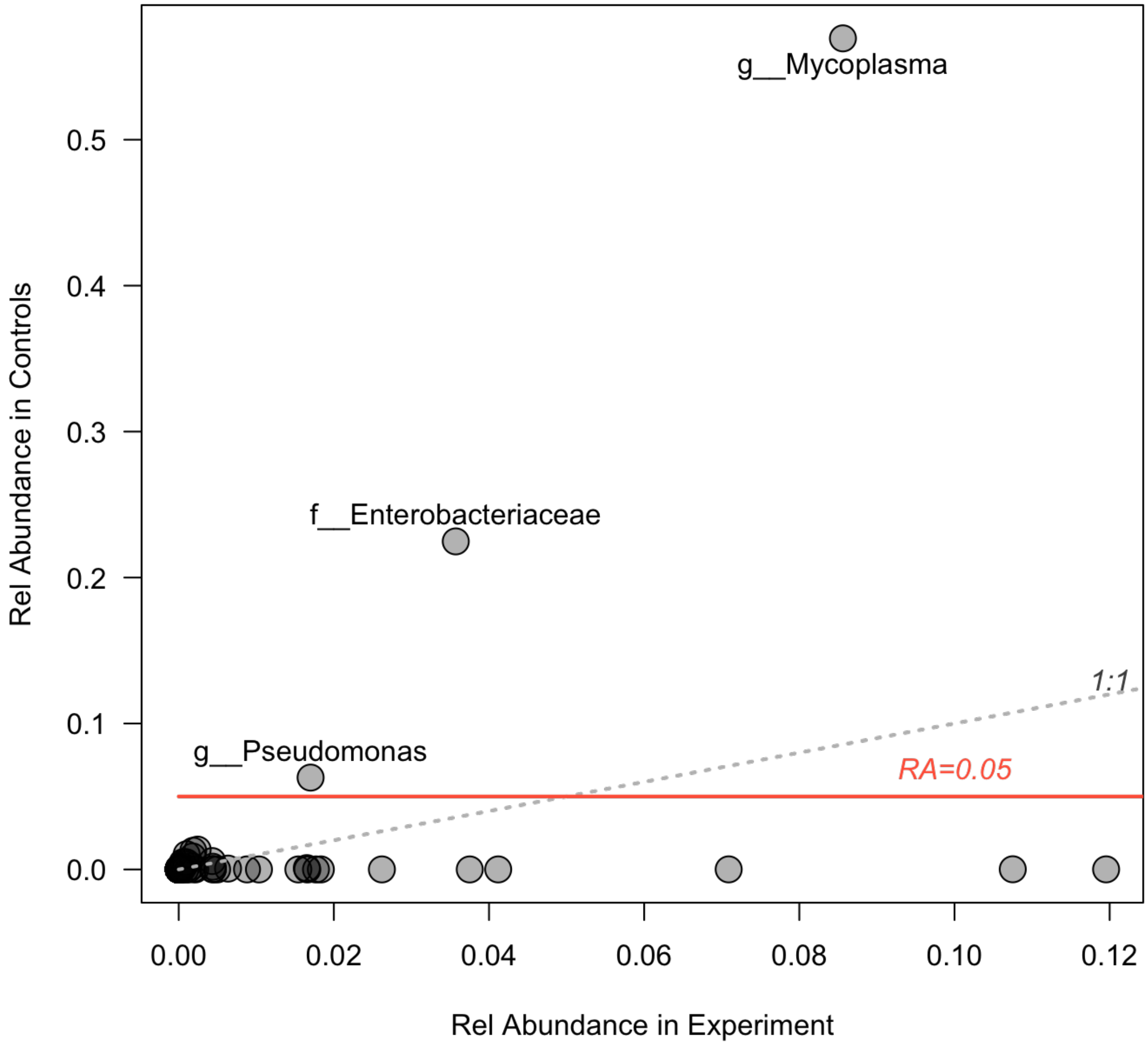

Figure 1: plot of chunk plotContam

Canberra distance Ok. During peer review, there was a question about how important contaminants were, and if useful information is lost by removing them. We removed them all the first time around, but this is a test to determine how consequential that overly conservative decision was. Pretty much all important results relied on the Jaccard and Canberra distances. So this approach will check the correlation of these matrices to determine whether we gain much information by including potential contaminants. Based on these results, we've elected to remove only the three OTUs that were clearly abundant in lab controls, instead of those that were more abundant in experimental samples.

Here are a few important metrics to consider:

```
allOTUs <- colnames(rw.table.allControl)[which(colSums(rw.table.allControl) > 0)]  # names of all otus
percentOTUsAlsoExp <- length(intersect(cont.otus.names, allOTUs)) / length(allOTUs) * 100
finalOTUs <- colnames(rw.25.ALL)[which(colSums(rw.25.ALL) > 0)]
numberInFinal <- length(intersect(cont.otus.names, finalOTUs))
percentInFinal <- length(intersect(cont.otus.names, finalOTUs)) / length(allOTUs) * 100
```

So only three contaminants are actually highly abundant in the controls and in experimental samples. In all, 3.4244 percent of OTUs (1178 total) were removed in the most conservative approach. And about 10.9928% were also in experimental samples (i.e., not just in control samples). If all contaminants would have been kept through analysis, rarefaction, and other trimming, 7.6771% (815 OTUs) of the final would have been from contaminants.

Here is the influence on beta-diversity from these contaminants using the Jaccard Distance:

```
dists.jac <- data.frame(No_Contam = as.vector(jac.NC), Minus_3_Contam = as.vector(jac.N3),
  All_Contam = as.vector(jac.ALL))
source('ggcorplot.R')
ggcorplot(
  data=dists.jac,
    var_text_size=5)
```

And here is a look at how the Canberra metric was influenced by removing contaminants:

```
dists.can <- data.frame(No_Contam = as.vector(can.NC), Minus_3_Contam = as.vector(can.N3),
  All_Contam = as.vector(can.ALL))
ggcorplot(
  data=dists.can,
  var_text_size=5)
```

Both of the distance matrices indicate that removing just the three most offensive OTUs (`Minus_3_contam`) and leaving all three potential contaminants in the dataset (`All_Contam`) give a very similar answer by virtue of a relatively high correlations. But removing all potential contaminants (`No_Contam`) results in a lower correlation to both other approaches. This seems to indicate that the three most obvious contaminants did indeed influence the results, but quite a bit of beta-diversity influence also came from the other OTUs that were much more rare in control samples. It also appears that some of the OTUs removed in the `No_Contam` approach were indeed some very common and important human-associated bacteria. So in the final version of the manusrcipt, we elected to remove the 3 most obvious contaminants and leave the rest.

```
ls()
```

```
 [1] "allOTUs"                    "can.ALL"
 [3] "can.N3"                     "can.NC"
 [5] "cont"                       "cont.otus"
```

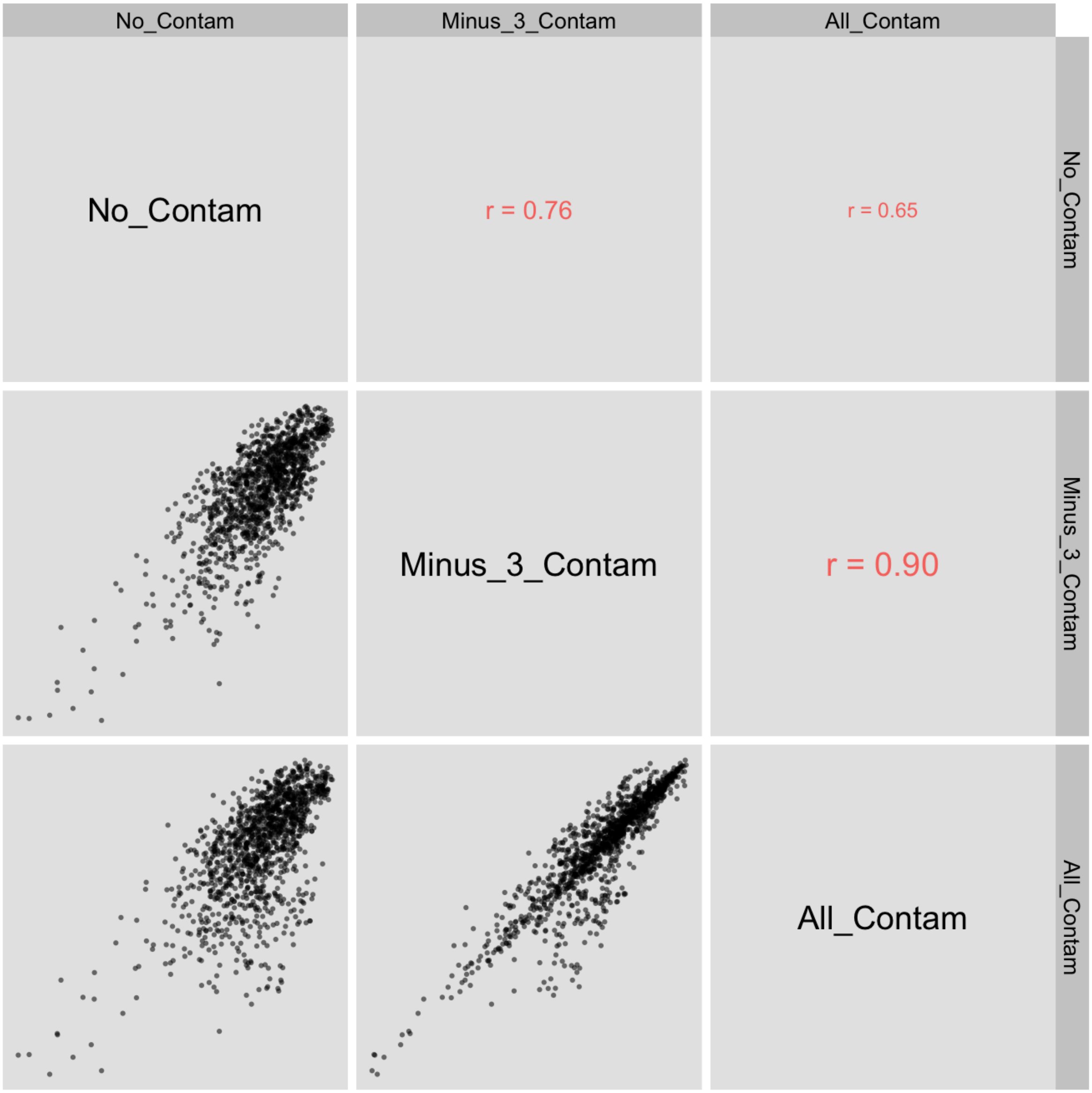

Figure 2: plot of chunk regressJacControlCheck

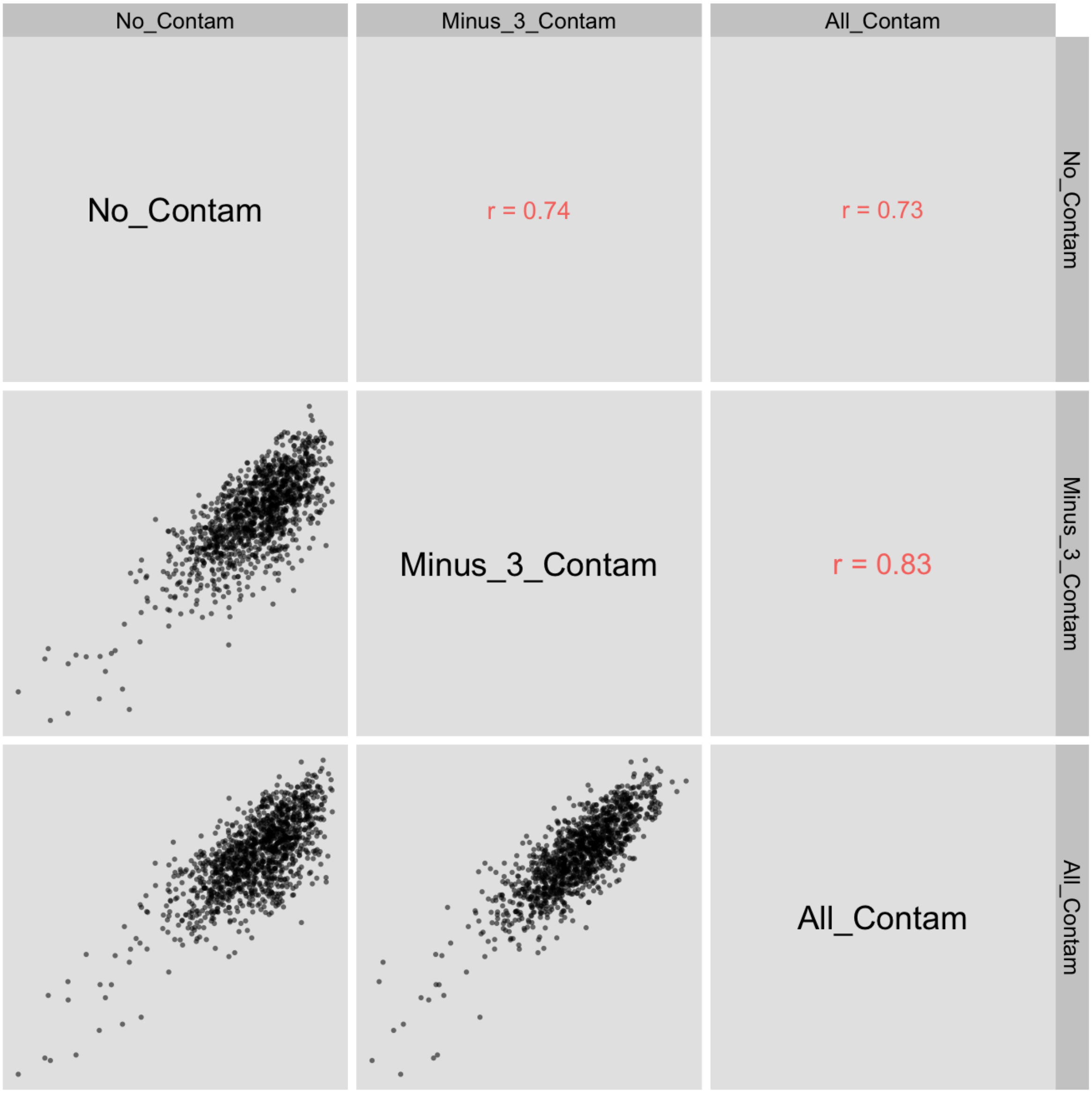

Figure 3: plot of chunk regressCanberraControlCheck

```
 [7] "cont.otus.names"        "cont.table"
 [9] "cont.taxo"              "cont3.otus"
[11] "dists.can"              "dists.jac"
[13] "Evenness"               "ezLev"
[15] "finalOTUs"              "ggcorplot"
[17] "jac.ALL"                "jac.N3"
[19] "jac.NC"                 "makeTaxo"
[21] "mitochondria"           "numberInFinal"
[23] "percentInFinal"         "percentOTUsAlsoExp"
[25] "plotX"                  "plotY"
[27] "QiimeIn"                "rw.25.ALL"
[29] "rw.25.N3"               "rw.25.NC"
[31] "rw.big"                 "rw.map"
[33] "rw.table.allControl"    "rw.table.nocontrol"
[35] "rw.table.noTop3control" "rw.table.tmp"
[37] "rw.taxo"                "rw.taxo.ALL"
[39] "rw.taxo.N3"             "rw.taxo.NC"
[41] "rw.taxo.tmp"            "streptophyta"
```

```
save.image('phones_contam_knitr.RData')
```
